# Phylogenomics of the Reproductive Parasite *Wolbachia pipientis w*Mel: A Streamlined Genome Overrun by Mobile Genetic Elements

**DOI:** 10.1371/journal.pbio.0020069

**Published:** 2004-03-16

**Authors:** Martin Wu, Ling V Sun, Jessica Vamathevan, Markus Riegler, Robert Deboy, Jeremy C Brownlie, Elizabeth A McGraw, William Martin, Christian Esser, Nahal Ahmadinejad, Christian Wiegand, Ramana Madupu, Maureen J Beanan, Lauren M Brinkac, Sean C Daugherty, A. Scott Durkin, James F Kolonay, William C Nelson, Yasmin Mohamoud, Perris Lee, Kristi Berry, M. Brook Young, Teresa Utterback, Janice Weidman, William C Nierman, Ian T Paulsen, Karen E Nelson, Hervé Tettelin, Scott L O'Neill, Jonathan A Eisen

**Affiliations:** **1**The Institute for Genomic Research, RockvilleMarylandUnited States of America; **2**Department of Epidemiology and Public Health, Yale University School of MedicineNew Haven, ConnecticutUnited States of America; **3**Department of Zoology and Entomology, School of Life SciencesThe University of Queensland, St Lucia, QueenslandAustralia; **4**Institut für Botanik III, Heinrich-Heine UniversitätDüsseldorfGermany

## Abstract

The complete sequence of the 1,267,782 bp genome of *Wolbachia pipientis w*Mel, an obligate intracellular bacteria of Drosophila melanogaster, has been determined. *Wolbachia*, which are found in a variety of invertebrate species, are of great interest due to their diverse interactions with different hosts, which range from many forms of reproductive parasitism to mutualistic symbioses. Analysis of the *w*Mel genome, in particular phylogenomic comparisons with other intracellular bacteria, has revealed many insights into the biology and evolution of *w*Mel and *Wolbachia* in general. For example, the *w*Mel genome is unique among sequenced obligate intracellular species in both being highly streamlined and containing very high levels of repetitive DNA and mobile DNA elements. This observation, coupled with multiple evolutionary reconstructions, suggests that natural selection is somewhat inefficient in *w*Mel, most likely owing to the occurrence of repeated population bottlenecks. Genome analysis predicts many metabolic differences with the closely related *Rickettsia* species, including the presence of intact glycolysis and purine synthesis, which may compensate for an inability to obtain ATP directly from its host, as *Rickettsia* can. Other discoveries include the apparent inability of *w*Mel to synthesize lipopolysaccharide and the presence of the most genes encoding proteins with ankyrin repeat domains of any prokaryotic genome yet sequenced. Despite the ability of *w*Mel to infect the germline of its host, we find no evidence for either recent lateral gene transfer between *w*Mel and D. melanogaster or older transfers between *Wolbachia* and any host. Evolutionary analysis further supports the hypothesis that mitochondria share a common ancestor with the α-Proteobacteria, but shows little support for the grouping of mitochondria with species in the order Rickettsiales. With the availability of the complete genomes of both species and excellent genetic tools for the host, the *w*Mel–D. melanogaster symbiosis is now an ideal system for studying the biology and evolution of *Wolbachia* infections.

## Abstract 2

The genome sequence of *Wolbachia* provides insights into the origins of mitochondria, as well as the ecology and evolution of endosymbiosis

## Introduction 

*Wolbachia* are intracellular gram-negative bacteria that are found in association with a variety of invertebrate species, including insects, mites, spiders, terrestrial crustaceans, and nematodes. *Wolbachia* are transovarialy transmitted from females to their offspring and are extremely widespread, having been found to infect 20%–75% of invertebrate species sampled (Jeyaprakash and Hoy 2000; Werren and Windsor 2000). *Wolbachia* are members of the Rickettsiales order of the α-subdivision of the Proteobacteria phyla and belong to the Anaplasmataceae family, with members of the genera *Anaplasma*, *Ehrlichia*, *Cowdria*, and *Neorickettsia* (Dumler et al. 2001). Six major clades (A–F) of *Wolbachia* have been identified to date (Lo et al. 2002): A, B, E, and F have been reported from insects, arachnids, and crustaceans; C and D from filarial nematodes.

*Wolbachia–*host interactions are complex and range from mutualistic to pathogenic, depending on the combination of host and *Wolbachia* involved. Most striking are the various forms of “reproductive parasitism” that serve to alter host reproduction in order to enhance the transmission of this maternally inherited agent. These include parthenogenesis (infected females reproducing in the absence of mating to produce infected female offspring), feminization (infected males being converted into functional phenotypic females), male-killing (infected male embryos being selectively killed), and cytoplasmic incompatibility (in its simplest form, the developmental arrest of offspring of uninfected females when mated to infected males) (O'Neill et al. 1997a).

*Wolbachia* have been hypothesized to play a role in host speciation through the reproductive isolation they generate in infected hosts (Werren 1998). They also provide an intriguing array of evolutionary solutions to the genetic conflict that arises from their uniparental inheritance. These solutions represent alternatives to classical mutualism and are often of more benefit to the symbiont than the host that is infected (Werren and O'Neill 1997). From an applied perspective, it has been proposed that *Wolbachia* could be utilized to either suppress pest insect populations or sweep desirable traits into pest populations (e.g., the inability to transmit disease-causing pathogens) (Sinkins and O'Neill 2000). Moreover, they may provide a new approach to the control of human and animal filariasis. Since the nematode worms that cause filariasis have an obligate symbiosis with mutualistic *Wolbachia*, treatment of filariasis with simple antibiotics that target *Wolbachia* has been shown to eliminate microfilaria production as well as ultimately killing the adult worm (Taylor et al. 2000; Taylor and Hoerauf 2001).

Despite their common occurrence and major effects on host biology, little is currently known about the molecular mechanisms that mediate the interactions between *Wolbachia* and their invertebrate hosts. This is partly due to the difficulty of working with an obligate intracellular organism that is difficult to culture and hard to obtain in quantity. Here we report the completion and analysis of the genome sequence of Wolbachia pipientis *w*Mel, a strain from the A supergroup that naturally infects Drosophila melanogaster (Zhou et al. 1998).

## Results/Discussion

### Genome Properties

**Figure 1:**
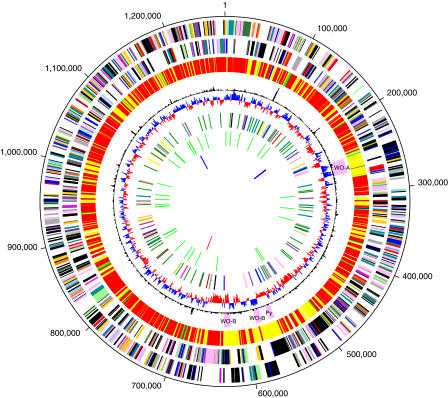
Circular Map of the Genome and Genome Features Circles correspond to the following: (1) forward strand genes; (2) reverse strand genes, (3) in red, genes with likely orthologs in both R. conorii and R. prowazekii; in blue, genes with likely orthologs in R. prowazekii, but absent from R. conorii; in green, genes with likely orthologs in R. conorii but absent from R. prowazekii; in yellow, genes without orthologs in either *Rickettsia* (Table S3); (4) plot is of χ^2^ analysis of nucleotide composition; phage regions are in pink; (5) plot of GC skew (G–C)/(G+C); (6) repeats over 200 bp in length, colored by category; (7) in green, transfer RNAs; (8) in blue, ribosomal RNAs; in red, structural RNA.

The *w*Mel genome is determined to be a single circular molecule of 1,267,782 bp with a G+C content of 35.2%. This assembly is very similar to the genetic and physical map of the closely related strain *w*MelPop (Sun et al., 2003). The genome does not exhibit the GC skew pattern typical of some prokaryotic genomes (Figure 1) that have two major shifts, one near the origin and one near the terminus of replication. Therefore, identification of a putative origin of replication and the assignment of basepair 1 were based on the location of the *dnaA* gene. Major features of the genome and of the annotation are summarized in Table 1 and Figure 1.

**Table 1:**
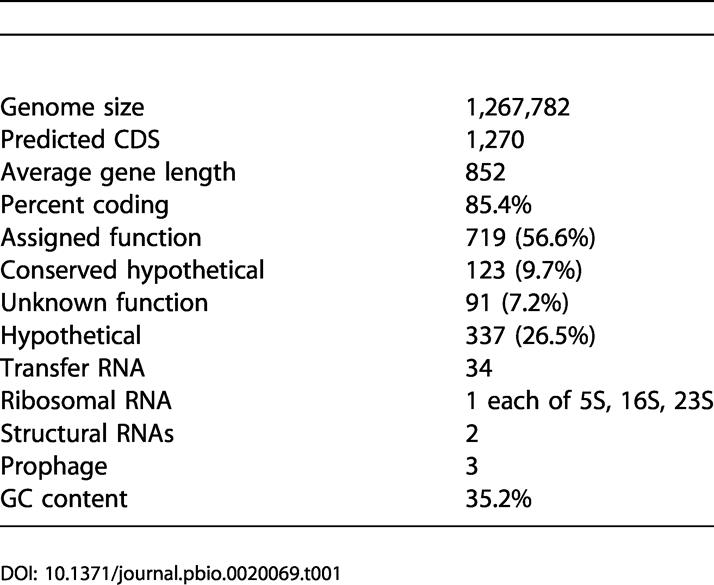
*w*Mel Genome Features

### Repetitive and Mobile DNA

The most striking feature of the *w*Mel genome is the presence of very large amounts of repetitive DNA and DNA corresponding to mobile genetic elements, which is unique for an intracellular species. In total, 714 repeats of greater than 50 bp in length, which can be divided into 158 distinct families (Table S1), were identified. Most of the repeats are present in only two copies in the genome, although 39 are present in three or more copies, with the most abundant repeat being found in 89 copies. We focused our analysis on the 138 repeats of greater than 200 bp (Table 2). These were divided into 19 families based upon sequence similarity to each other. These repeats were found to make up 14.2 % of the *w*Mel genome. Of these repeat families, 15 correspond to likely mobile elements, including seven types of insertion sequence (IS) elements, four likely retrotransposons, and four families without detectible similarity to known elements but with many hallmarks of mobile elements (flanked by inverted repeats, present in multiple copies) (Table 2). One of these new elements (repeat family 8) is present in 45 copies in the genome. It is likely that many of these elements are not able to autonomously transpose since many of the transposase genes are apparently inactivated by mutations or the insertion of other transposons (Table S2). However, some are apparently recently active since there are transposons inserted into at least nine genes (Table S2), and the copy number of some repeats appears to be variable between *Wolbachia* strains (M. Riegler et al., personal communication). Thus, many of these repetitive elements may be useful markers for strain discrimination. In addition, the mobile elements likely contribute to generating the diversity of phenotypically distinct *Wolbachia* strains (e.g., mod^−^ strains [McGraw et al. 2001]) by altering or disrupting gene function (Table S2).

**Table 2:**
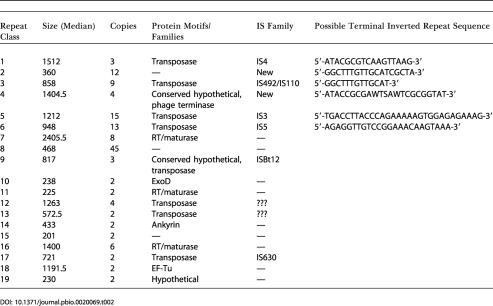
*w*Mel DNA Repeats of Greater than 200 bp

**Figure 2:**
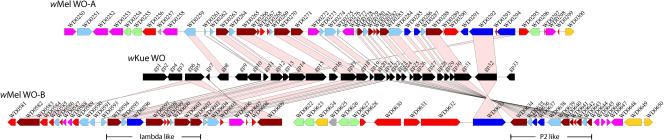
Phage Alignments and Neighboring Genes Conserved gene order between the WO phage in *Wolbachia* sp. *w*Kue and prophage regions of *w*Mel. Putative proteins in *w*Kue (Masui et al. 2001) were searched using TBLASTN against the *w*Mel genome. Matches with an *E*-value of less than 1e^−15^ are linked by connecting lines. CDSs are colored as follows: brown, phage structural or replication genes; light blue, conserved hypotheticals; red, hypotheticals; magenta, transposases or reverse transcriptases; blue, ankyrin repeat genes; light gray, *radC*; light green, paralogous genes; gold, others. The regions surrounding the phage are shown because they have some unusual features relative to the rest of the genome. For example, WO-A and WO-B are each flanked on one side by clusters of genes in two paralogous families that are distantly related to phage repressors. In each of these clusters, a homolog of the *radC* gene is found. A third *radC* homolog (WD1093) in the genome is also flanked by a member of one of these gene families (WD1095). While the connection between *radC* and the phage is unclear, the multiple copies of the *radC* gene and the members of these paralogous families may have contributed to the phage rearrangements described above.

Three prophage elements are present in the genome. One is a small pyocin-like element made up of nine genes (WD00565–WD00575). The other two are closely related to and exhibit extensive gene order conservation with the WO phage described from *Wolbachia* sp. *w*Kue (Masui et al. 2001) (Figure 2). Thus, we have named them *w*Mel WO-A and WO-B, based upon their location in the genome. *w*Mel WO-B has undergone a major rearrangement and translocation, suggesting it is inactive. Phylogenetic analysis indicates that *w*Mel WO-B is more closely related to the *w*Kue WO than to *w*Mel WO-A (Figure S1). Thus, *w*Mel WO-A likely represents either a separate insertion event in the *Wolbachia* lineage or a duplication that occurred prior to the separation of the *w*Mel and *w*Kue lineages. Phylogenetic analysis also confirms the proposed mosaic nature of the WO phage (Masui et al. 2001), with one block being closely related to lambdoid phage and another to P2 phage (data not shown).

### Genome Structure: Rearrangements, Duplications, and Deletions

**Figure 3:**
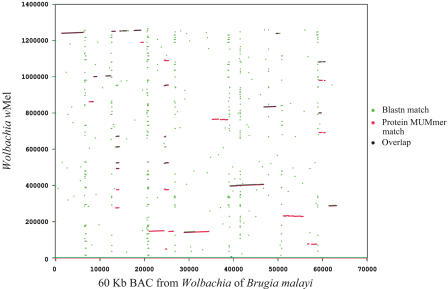
Alignment of *w*Mel with a 60 kbp Region of the *Wolbachia* from *B. malayi* The figure shows BLASTN matches (green) and whole-proteome alignments (red) that were generated using the “promer” option of the MUMmer software (Delcher et al. 1999). The B. malayi region is from a BAC clone (Ware et al. 2002). Note the regions of alignment broken up by many rearrangements and the presence of repetitive sequences at the regions of the breaks.

The irregular pattern of GC skew in *w*Mel is likely due in part to intragenomic rearrangements associated with the many DNA repeat elements. Comparison with a large contig from a *Wolbachia* species that infects Brugia malayi is consistent with this (Ware et al. 2002) (Figure 3). While only translocations are seen in this plot, genetic comparisons reveal that inversions also occur between strains (Sun et al., 2003), which is consistent with previous studies of prokaryotic genomes that have found that the most common large-scale rearrangements are inversions that are symmetric around the origin of DNA replication (Eisen et al. 2000). The occurrence of frequent rearrangement events during *Wolbachia* evolution is supported by the absence of any large-scale conserved gene order with *Rickettsia* genomes. The rearrangements in *Wolbachia* likely correspond with the introduction and massive expansion of the repeat element families that could serve as sites for intragenomic recombination, as has been shown to occur for some other bacterial species (Parkhill et al. 2003). The rearrangements in *w*Mel may have fitness consequences since several classes of genes often found in clusters are generally scattered throughout the *w*Mel genome (e.g., ABC transporter subunits, Sec secretion genes, rRNA genes, F-type ATPase genes).

Although the common ancestor of *Wolbachia* and *Rickettsia* likely already had a reduced, streamlined genome, *w*Mel has lost additional genes since that time (Table S3). Many of these recent losses are of genes involved in cell envelope biogenesis in other species, including most of the machinery for producing lipopolysaccharide (LPS) components and the alanine racemase that supplies D-alanine for cell wall synthesis. In addition, some other genes that may have once been involved in this process are present in the genome, but defective (e.g., mannose-1-phosphate guanylyltransferase, which is split into two coding sequences [CDSs], WD1224 and WD1227, by an IS5 element) and are likely in the process of being eliminated. The loss of cell envelope biogenesis genes has also occurred during the evolution of the *Buchnera* endosymbionts of aphids (Shigenobu et al. 2000; Moran and Mira 2001). Thus, *w*Mel and *Buchnera* have lost some of the same genes separately during their reductive evolution. Such convergence means that attempts to use gene content to infer evolutionary relatedness needs to be interpreted with caution. In addition, since *Anaplasma* and *Ehrlichia* also apparently lack genes for LPS production (Lin and Rikihisha 2003), it is likely that the common ancestor of *Wolbachia*, *Ehrlichia*, and *Anaplasma* was unable to synthesize LPS. Thus, the reports that *Wolbachia*-derived LPS-like compounds is involved in the immunopathology of filarial nematode disease in mammals (Taylor 2002) either indicate that these *Wolbachia* have acquired genes for LPS synthesis or that the reported LPS-like compounds are not homologous to LPS.

Despite evident genome reduction in *w*Mel and in contrast to most small-genomed intracellular species, gene duplication appears to have continued, as over 50 gene families have apparently expanded in the *w*Mel lineage relative to that of all other species (Table S4). Many of the pairs of duplicated genes are encoded next to each other in the genome, suggesting that they arose by tandem duplication events and may simply reflect transient duplications in evolution (deletion is common when there are tandem arrays of genes). Many others are components of mobile genetic elements, indicating that these elements have expanded significantly after entering the *Wolbachia* evolutionary lineage. Other duplications that could contribute to the unique biological properties of *w*Mel include that of the mismatch repair gene *mutL* (see below) and that of many hypothetical and conserved hypothetical proteins.

One duplication of particular interest is that of *wsp*, which is a standard gene for strain identification and phylogenetic reconstruction in *Wolbachia* (Zhou et al. 1998). In addition to the previously described *wsp* (WD0159), *w*Mel encodes two *wsp* paralogs (WD0009 and WD0489), which we designate as *wspB* and *wspC*, respectively. While these paralogs are highly divergent from *wsp* (protein identities of 19.7% and 23.5%, respectively) and do not amplify using the standard *wsp* PCR primers (Braig et al. 1998; Zhou et al. 1998), their presence could lead to some confusion in classification and identification of *Wolbachia* strains. This has apparently occurred in one study of *Wolbachia* strain *w*KueYO, for which the reported *wsp* gene (gbAB045235) is actually an ortholog of *wspB* (99.8% sequence identity and located at the end of the *virB* operon [Masui et al. 2000]) and not an ortholog of the *wsp* gene. Considering that the *wsp* gene has been extremely informative for discriminating between strains of *Wolbachia*, we designed PCR primers to the *w*Mel *wspB* gene to amplify and then sequence the orthologs from the related *w*Ri and *w*AlbB *Wolbachia* strains from Drosophila simulans and Aedes albopictus, respectively, as well as the *Wolbachia* strain that infects the filarial nematode Dirofilaria immitis to determine the potential utility of this locus for strain discrimination. A comparison of genetic distances between the *wsp* and *wspB* genes for these different taxa indicates that overall the *wspB* gene appears to be evolving at a faster rate than *wsp* and, as such, may be a useful additional marker for discriminating between closely related *Wolbachia* strains (Table S5).

### Inefficiency of Selection in *w*Mel

The fraction of the genome that is repetitive DNA and the fraction that corresponds to mobile genetic elements are among the highest for any prokaryotic genome. This is particularly striking compared to the genomes of other obligate intracellular species such as *Buchnera*, *Rickettsia*, *Chlamydia*, and *Wigglesworthia*, that all have very low levels of repetitive DNA and mobile elements. The recently sequenced genomes of the intracellular pathogen Coxiella burnetti (Seshadri et al. 2003) has both a streamlined genome and moderate amounts of repetitive DNA, although much less than *w*Mel. The paucity of repetitive DNA in these and other intracellular species is thought to be due to a combination of lack of exposure to other species, thereby limiting introduction of mobile elements, and genome streamlining (Mira et al. 2001; Moran and Mira 2001; Frank et al. 2002). We examined the *w*Mel genome to try to understand the origin of the repetitive and mobile DNA and to explain why such repetitive/mobile DNA is present in *w*Mel, but not other streamlined intracellular species.

We propose that the mobile DNA in *w*Mel was acquired some time after the separation of the *Wolbachia* and *Rickettsia* lineages but before the radiation of the *Wolbachia* group*.* The acquisition of these elements after the separation of the *Wolbachia* and *Rickettsia* lineages is suggested by the fact that most do not have any obvious homologous sequences in the genomes of other α-Proteobacteria, including the closely related *Rickettsia* spp. Additional evidence for some acqui-sition of foreign DNA after the *Wolbachia–Rickettsia* split comes from phylogenetic analysis of those genes present in *w*Mel, but not in the two sequenced rickettsial genomes (see Table S3; unpublished data). The acquisition prior to the radiation of *Wolbachia* is suggested by two lines of evidence. First, many of the elements are found in the genome of the distantly related *Wolbachia* of the nematode B. malayi (see Figure 3; unpublished data). In addition, genome analysis reveals that these elements do not have significantly anomalous nucleotide composition or codon usage compared to the rest of the genome. In fact, there are only four regions of the genome with significantly anomalous composition, comprising in total only approximately 17 kbp of DNA (Table 3). The lack of anomalous composition suggests either that any foreign DNA in *w*Mel was acquired long enough ago to allow it to “ameliorate” and become compositionally similar to endogenous *Wolbachia* DNA (Lawrence and Ochman 1997, 1998) or that any foreign DNA that is present was acquired from organisms with similar composition to endogenous *w*Mel genes. Owing to their potential effects on genome evolution (insertional mutagenesis, catalyzing genome rearrangements), we propose that the acquisition and maintenance of these repetitive and mobile elements by *w*Mel have played a key role in shaping the evolution of *Wolbachia*.

**Table 3:**
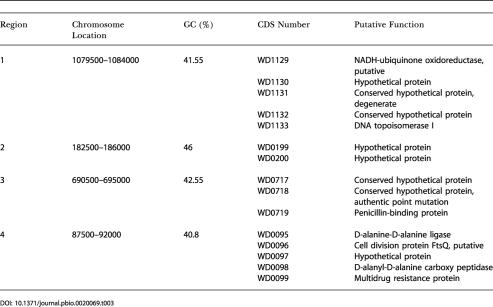
Regions of Anomalous Nucleotide Composition in the *wMel* Genome

**Figure 4:**
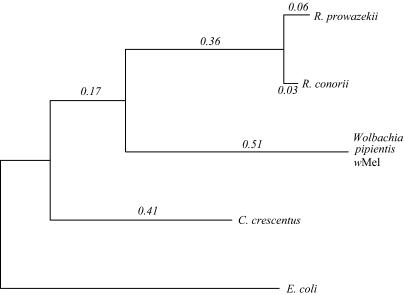
Long Evolutionary Branches in *w*Mel Maximum-likelihood phylogenetic tree constructed on concatenated protein sequences of 285 orthologs shared among *w*Mel, R. prowazekii, R. conorii, *C. crescentus,* and E. coli. The location of the most recent common ancestor of the α-Proteobacteria (*Caulobacter*, *Rickettsia*, *Wolbachia*) is defined by the outgroup *E. coli.* The unit of branch length is the number of changes per amino acid. Overall, the amino acid substitution rate in the *w*Mel lineage is about 63% higher than that of *C. crescentus*, a free-living α-Proteobacteria. *w*Mel has evolved at a slightly higher rate than the *Rickettssia* spp., close relatives that are also obligate intracellular bacteria that have undergone accelerated evolution themselves. This higher rate is likely in part to be due to an increase in the rate of slightly deleterious mutations, although we have not ruled out the possibility of G+C content effects on the branch lengths.

It is likely that much of the mobile/repetitive DNA was introduced via phage, given that three prophage elements are present; experimental studies have shown active phage in some *Wolbachia* (Masui et al. 2001) and *Wolbachia* superinfections occur in many hosts (e.g., Jamnongluk et al. 2002), which would allow phage to move between strains. Whatever the mechanism of introduction, the persistence of the repetitive elements in *w*Mel in the face of apparently strong pressures for streamlining is intriguing. One expla-nation is that *w*Mel may be getting a steady infusion of mobile elements from other *Wolbachia* strains to counteract the elimination of elements by selection for genome streamlining. This would explain the absence of anomalous nucleotide composition of the elements. However, we believe that a major contributing factor to the presence of all the repetitive/mobile DNA in *w*Mel is that *w*Mel and possibly *Wolbachia* in general have general inefficiency of natural selection relative to other species. This inefficiency would limit the ability to eliminate repetitive DNA. A general inefficiency of natural selection (especially purifying selection) has been suggested previously for intracellular bacteria, based in part on observations that these bacteria have higher evolutionary rates than free-living bacteria (e.g., Moran 1996). We also find a higher evolutionary rate for *w*Mel than that of the closely related intracellular *Rickettsia*, which themselves have higher rates than free-living α-Proteobacteria (Figure 4). Additionally, codon bias in *w*Mel appears to be driven more by mutation or drift than selection (Figure S2), as has been reported for *Buchnera* species and was suggested to be due to inefficient purifying selection (Wernegreen and Moran 1999). Such inefficiencies of natural selection are generally due to an increase in the relative contribution of genetic drift and mutation as compared to natural selection (Eiglmeier et al. 2001; Lawrence 2001; Parkhill et al. 2001). Below we discuss different possible explanations for the inefficiency of selection in *w*Mel, especially in comparison to other intracellular bacteria.

Low rates of recombination, such as occur in centromeres and the human Y chromosome, can lead to inefficient selection because of the linkage among genes. This has been suggested to be occurring in *Buchnera* species because these species do not encode homologs of RecA, which is the key protein in homologous recombination in most species (Shigenobu et al. 2000). The absence of recombination in *Buchnera* is supported by the lack of genome rearrangements in their recent evolution (Tamas et al. 2002). Additionally, there is apparently little or no gene flow into *Buchnera* strains. In contrast, *w*Mel encodes the necessary machinery for recombination, including RecA (Table S6), and has experienced both extensive intragenomic homologous recombination and introduction of foreign DNA. Therefore, the unusual genome features of *w*Mel are unlikely to be due to low levels of recombination.

Another possible explanation for inefficient selection is high mutation rates. It has been suggested that the higher evolutionary rates in intracellular bacteria are the result of high mutation rates that are in turn due to the loss of genes for DNA repair processes (e.g., Itoh et al. 2002). This is likely not the case in *w*Mel since its genome encodes proteins corresponding to a broad suite of DNA repair pathways including mismatch repair, nucleotide excision repair, base excision repair, and homologous recombination (Table S6). The only noteworthy DNA repair gene absent from *w*Mel and present in the more slowly evolving *Rickettsia* is *mfd,* which is involved in targeting DNA repair to the transcribed strand of actively transcribing genes in other species (Selby et al. 1991). However, this absence is unlikely to contribute significantly to the increased evolutionary rate in *w*Mel, since defects in *mfd* do not lead to large increases in mutation rates in other species (Witkin 1994). The presence of mismatch repair genes (homologs of *mutS* and *mutL*) in *w*Mel is particularly relevant since this pathway is one of the key steps in regulating mutation rates in other species. In fact, *w*Mel is the first bacterial species to be found with two *mutL* homologs. Overall, examination of the predicted DNA repair capabilities of bacteria (Eisen and Hanawalt 1999) suggests that the connection between evolutionary rates in intracellular species and the loss of DNA repair processes is spurious. While many intracellular species have lost DNA repair genes in their recent evolution, different species have lost different genes and some, such as *w*Mel and *Buchnera* spp., have kept the genes that likely regulate mutation rates. In addition, some free-living species without high evolutionary rates have lost some of the same pathways lost in intracellular species, while many free-living species have lost key pathways resulting in high mutation rates (e.g., Helicobacter pylori has apparently lost mismatch repair [Eisen 1997, Eisen 1998b; Bjorkholm et al. 2001]). Given that intracellular species tend to have small genomes and have lost genes from every type of biological process, it is not surprising that many of them have lost DNA repair genes as well.

We believe that the most likely explanations for the inefficiency of selection in *w*Mel involve population-size related factors, such as genetic drift and the occurrence of population bottlenecks. Such factors have also been shown to likely explain the high evolutionary rates in other intracellular species (Moran 1996; Moran and Mira 2001; van Ham et al. 2003). *Wolbachia* likely experience frequent population bottlenecks both during transovarial transmission (Boyle et al. 1993) and during cytoplasmic incompatibility mediated sweeps through host populations. The extent of these bottlenecks may be greater than in other intracellular bacteria, which would explain why *w*Mel has both more repetitive and mobile DNA than other such species and a higher evolutionary rate than even the related *Rickettsia spp.* Additional genome sequences from other *Wolbachia* will reveal whether this is a feature of all *Wolbachia* or only certain strains.

### Mitochondrial Evolution

There is a general consensus in the evolutionary biology literature that the mitochondria evolved from bacteria in the α-subgroup of the Proteobacteria phyla (e.g., Lang et al. 1999). Analysis of complete mitochondrial and bacterial genomes has very strongly supported this hypothesis (Andersson et al. 1998, 2003; Muller and Martin 1999; Ogata et al. 2001). However, the exact position of the mitochondria within the α-Proteobacteria is still debated. Many studies have placed them in or near the Rickettsiales order (Viale and Arakaki 1994; Gupta 1995; Sicheritz-Ponten et al. 1998; Lang et al. 1999; Bazinet and Rollins 2003). Some studies have further suggested that mitochondria are a sister taxa to the *Rickettsia* genus within the Rickettsiaceae family and thus more closely related to *Rickettsia* spp. than to species in the Anaplasmataceae family such as *Wolbachia* (Karlin and Brocchieri 2000; Emelyanov 2001a, 2001b, 2003a, 2003b).

**Figure 5:**
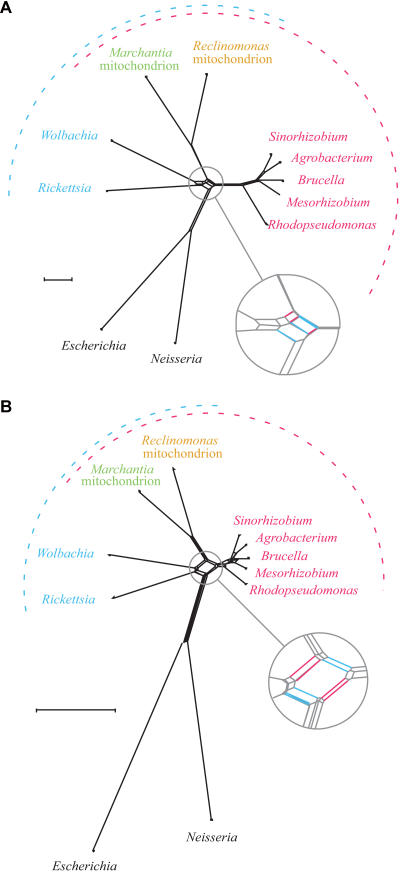
Mitochondrial Evolution Using Concatenated Alignments Networks of protein LogDet distances for an alignment of 32 proteins constructed with Neighbor-Net (Bryant and Moulton 2003). The scale bar indicates 0.1 substitutions per site. Enlargements at lower right show the component of shared similarity between mitochondrial-encoded proteins and (i) their homologs from intracellular endosymbionts (red) as well as (ii) their homologs from free-living α-Proteobacteria (blue). (A) Result using 6,776 gap-free sites per genome (heavily biased in amino acid composition). (B) Result using 3,100 sites after exclusion of highly variable positions (data not biased in amino acid composition at *p* = 0.95). All data and alignments are available upon request. Results of phylogenetic analyses are summa-rized in Table S7. Since amino acid content bias was very severe in these datasets, protein LogDet analyses were also preformed. In neighbor-joining, parsimony, and maximum-likelihood trees generated from alignments both including and excluding highly biased positions (6,776 and 3,100 gap-free amino acid sites per genome, respectively), mitochondria usually branched basal to the *Wolbachia–Rickettsia* clade, but never specifically with *Rickettsia* (Table S7).

In our analysis of complete genomes, including that of *w*Mel, the first non-*Rickettsia* member of the Rickettsiales order to have its genome completed, we find support for a grouping of *Wolbachia* and *Rickettsia* to the exclusion of the mitochondria, but not for placing the mitochondria within the Rickettsiales order (Figure 5A and 5B; Table S7; Table S8). Specifically, phylogenetic trees of a concatenated alignment of 32 proteins show strong support with all methods (see Table S7) for common branching of: (i) mitochondria, (ii) *Rickettsia* with *Wolbachia*, (iii) the free-living α-Proteobacteria, and (iv) mitochondria within α-Proteobacteria. Since amino acid content bias was very severe in these datasets, protein LogDet analyses, which can correct for the bias, were also performed. In LogDet analyses of the concatenated protein alignment, both including and excluding highly biased positions, mitochondria usually branched basal to the *Wolbachia–Rickettsia* clade, but never specifically with *Rickettsia* (see Table S7). In addition, in phylogenetic studies of individual genes, there was no consistent phylogenetic position of mitochondrial proteins with any particular species or group within the α-Proteobacteria (see Table S8), although support for a specific branch uniting the two *Rickettsia* species with *Wolbachia* was quite strong. Eight of the proteins from mitochondrial genomes (YejW, SecY, Rps8, Rps2, Rps10, RpoA, Rpl15, Rpl32) do not even branch within the α-Proteobacteria, although these genes almost certainly were encoded in the ancestral mitochondrial genome (Lang et al. 1997).

This analysis of mitochondrial and α-Proteobacterial genes reinforces the view that ancient protein phylogenies are inherently prone to error, most likely because current models of phylogenetic inference do not accurately reflect the true evolutionary processes underlying the differences observed in contemporary amino acid sequences (Penny et al. 2001). These conflicting results regarding the precise position of mitochondria within the α-Proteobacteria can be seen in the high amount of networking in the Neighbor-Net graph of the analyses of the concatenated alignment shown in Figure 5. An important complication in studies of mitochondrial evolution lies in identifying “α-Proteobacterial” genes for comparison (Martin 1999). For example, in our analyses, proteins from *Magnetococcus* branched with other α-Proteobacterial homologs in only 17 of the 49 proteins studied, and in five cases they assumed a position basal to α-, β-, and γ-Proteobacterial homologs.

### Host–Symbiont Gene Transfers

Many genes that were once encoded in mitochondrial genomes have been transferred into the host nuclear genomes. Searching for such genes has been complicated by the fact that many of the transfer events happened early in eukaryotic evolution and that there are frequently extreme amino acid and nucleotide composition biases in mitochondrial genomes (see above). We used the *w*Mel genome to search for additional possible mitochondrial-derived genes in eukaryotic nuclear genomes. Specifically, we constructed phylogenetic trees for *w*Mel genes that are not in either *Rickettsia* genomes. Five new eukaryotic genes of possible mitochondrial origin were identified: three genes involved in de novo nucleotide biosynthesis (*purD*, *purM*, *pyrD*) and two conserved hypothetical proteins (WD1005, WD0724). The α-Proteobacterial origin of these genes suggests that at least some of the genes of the de novo nucleotide synthesis pathway in eukaryotes might have been laterally acquired from bacteria via the mitochondria. The presence of such genes in other Proteobacteria suggests that their absence from *Rickettsia* is due to gene loss (Gray et al. 2001). This finding supports the need for additional α-Proteobacterial genomes to identify mitochondrion-derived genes in eukaryotes.

While organelle to nuclear gene transfers are generally accepted, there is a great deal of controversy over whether other gene transfers have occurred from bacteria into animals. In particular, claims of transfer from bacteria into the human genome (Lander et al. 2001) were later shown to be false (Roelofs and Van Haastert 2001; Salzberg et al. 2001; Stanhope et al. 2001). *Wolbachia* are excellent candidates for such transfer events since they live inside the germ cells, which would allow lateral transfers to the host to be transmitted to subsequent host generations. Consistent with this, a recent study has shown some evidence for the presence of *Wolbachia-*like genes in a beetle genome (Kondo et al. 2002). The symbiosis between *w*Mel and D. melanogaster provides an ideal case to search for such transfers since we have the complete genomes of both the host and symbiont. Using BLASTN searches and MUMmer alignments, we did not find any examples of highly similar stretches of DNA shared between the two species. In addition, protein-level searches and phylogenetic trees did not identify any specific relationships between *w*Mel and D. melanogaster for any genes. Thus, at least for this host–symbiont association, we do not find any likely cases of recent gene exchange, with genes being maintained in both host and symbiont. In addition, in our phylogenetic analyses, we did not find any examples of *w*Mel proteins branching specifically with proteins from any invertebrate to the exclusion of other eukaryotes. Therefore, at least for the genes in *w*Mel, we do not find evidence for transfer of *Wolbachia* genes into any invertebrate genome.

### Metabolism and Transport

*w*Mel is predicted to have very limited capabilities for membrane transport, for substrate utilization, and for the biosynthesis of metabolic intermediates (Figure S3), similar to what has been seen in other intracellular symbionts and pathogens (Paulsen et al. 2000). Almost all of the identifiable uptake systems for organic nutrients in *w*Mel are for amino acids, including predicted transporters for proline, asparate/glutamate, and alanine. This pattern of transporters, coupled with the presence of pathways for the metabolism of the amino acids cysteine, glutamate, glutamine, proline, serine, and threonine, suggests that *w*Mel may obtain much of its energy from amino acids. These amino acids could also serve as material for the production of other amino acids. In contrast, carbohydrate metabolism in *w*Mel appears to be limited. The only pathways that appear to be complete are the tricarboxylic acid cycle, the nonoxidative pentose phosphate pathway, and glycolysis, starting with fructose-1,6-biphosphate. The limited carbohydrate metabolism is consistent with the presence of only one sugar phosphate transporter. *w*Mel can also apparently transport a range of inorganic ions, although two of these systems, for potassium uptake and sodium ion/proton exchange, are frameshifted. In the latter case, two other sodium ion/proton exchangers may be able to compensate for this defect.

Many of the predicted metabolic properties of *w*Mel, such as the focus on amino acid transport and the presence of limited carbohydrate metabolism, are similar to those found in *Rickettsia.* A major difference with the *Rickettsia* spp. is the absence of the ADP–ATP exchanger protein in *w*Mel. In *Rickettsia* this protein is used to import ATP from the host, thus allowing these species to be direct energy scavengers (Andersson et al. 1998). This likely explains the presence of glycolysis in *w*Mel but not *Rickettsia.* An inability to obtain ATP from its host also helps explain the presence of pathways for the synthesis of the purines AMP, IMP, XMP, and GMP in *w*Mel but not *Rickettsia.* Other pathways present in *w*Mel but not *Rickettsia* include threonine degradation (described above), riboflavin biosynthesis, pyrimidine metabolism (i.e., from PRPP to UMP), and chelated iron uptake (using a single ABC transporter). The two *Rickettsia* species have a relatively large complement of predicted transporters for osmoprotectants, such as proline and glycine betaine, whereas *w*Mel possesses only two of these systems.

### Regulatory Responses

The *w*Mel genome is predicted to encode few proteins for regulatory responses. Three genes encoding two-component system subunits are present: two sensor histidine kinases (WD1216 and WD1284) and one response regulator (WD0221). Only six strong candidates for transcription regulators were identified: a homolog of arginine repressors (WD0453), two members of the TenA family of transcription activator proteins (WD0139 and WD0140), a homolog of *ctrA*, a transcription regulator for two component systems in other α-Proteobacteria (WD0732), and two σ factors (RpoH/WD1064 and RpoD/WD1298). There are also seven members of one paralogous family of proteins that are distantly related to phage repressors (see above), although if they have any role in transcription, it is likely only for phage genes. Such a limited repertoire of regulatory systems has also been reported in other endosymbionts and has been explained by the apparent highly predictable and stable environment in which these species live (Andersson et al. 1998; Read et al. 2000; Shigenobu et al. 2000; Moran and Mira 2001; Akman et al. 2002; Seshadri et al. 2003).

### Host–Symbiont Interactions

**Figure 6:**
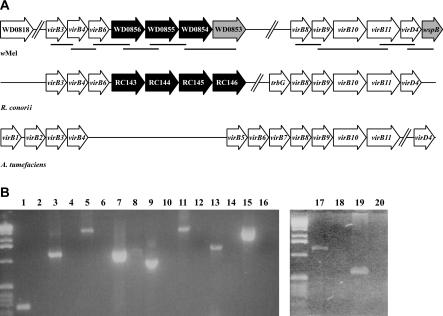
Genomic Organization and expression of Type IV Secretion Operons in *w*Mel (A) Organization of the nine *vir*-like CDSs (white arrows) and five adjacent CDSs that encode for either putative membrane-spanning proteins (black arrows) or non-*vir* CDSs (gray arrows) of wMel, R. conorii, and A. tumefaciens. Solid horizontal lines denote RT experiments that have confirmed that adjacent CDSs are expressed as part of a polycistronic transcript. Results of these RT-PCR experiments are presented in (B). Lane 1, *virB3*-*virB4*; lane 2, RT control; lane 3, *virB6*-WD0856; lane 4, RT control; lane 5, WD0856-WD0855; lane 6, RT control; lane 7, WD0854-WD0853; lane 8, RT control; lane 9, *virB8*-*virB9*; lane 10, RT control; lane 11, *virB9*-*virB11*; lane 12, RT control; lane 13, *virB11*-*virD4*; lane 14, RT control; lane 15, *virD4*-*wspB*; lane 16, RT control; lane 17, *virB4*-*virB6*; lane 18, RT control; lane 19, WD0855-WD0854; lane 20, RT control. Only PCRs that contain reverse transcriptase amplified the desired products. PCR primer sequences are listed in Table S9.

The mechanisms by which *Wolbachia* infect host cells and by which they cause the diverse phenotypic effects on host reproduction and fitness are poorly understood, and the *w*Mel genome helps identify potential contributing factors. A complete Type IV secretion system, portions of which have been reported in earlier studies, is present. The complete genome sequence shows that in addition to the five *vir* genes previously described from Wolbachia wKueYO (Masui et al. 2001), an additional four are present in *w*Mel. Of the nine *w*Mel *vir* ORFs, eight are arranged into two separate operons. Similar to the single operon identified in *w*Tai and *w*KueYO, the *w*Mel *virB8*, *virB9*, *virB10*, *virB11*, and *virD4* CDSs are adjacent to *wspB*, forming a 7 kb operon (WD0004–WD0009). The second operon contains *virB3*, *virB4*, and *virB6* as well as four additional non-*vir* CDSs, including three putative membrane-spanning proteins, that form part of a 15.7 kb operon (WD0859–WD0853). Examination of the Rickettsia conorii genome shows a similar orga-nization (Figure 6A). The observed conserved gene order for these genes between these two genomes suggests that the putative membrane-spanning proteins could form a novel and, possibly, integral part of a functioning Type IV secretion system within these bacteria. Moreover, reverse transcription (RT)-PCRs have confirmed that *wspB* and WD0853–WD0856 are each expressed as part of the two *vir* operons and further indicate that these additional encoded proteins are novel components of the *Wolbachia* Type IV secretion system (Figure 6B).

In addition to the two major *vir* clusters, a paralog of *virB8* (WD0817) is also present in the *w*Mel genome. WD0818 is quite divergent from *virB8* and, as such, does not appear to have resulted from a recent gene duplication event. RT-PCR experiments have failed to show expression of this CDS in *w*Mel-infected *Drosophila* (data not shown). PCR primers were designed to all CDSs of the *w*Mel Type IV secretion system and used to successfully amplify orthologs from the divergent *Wolbachia* strains *w*Ri and *w*AlbB (data not shown). We were able to detect orthologs to all of the *w*Mel Type IV secretion system components as well as most of the adjacent non-*vir* CDSs, suggesting that this system is conserved across a range of A- and B-group *Wolbachia*. An increasing body of evidence has highlighted the importance of Type IV secretion systems for the successful infection, invasion, and persistence of intracellular bacteria within their hosts (Christie 2001; Sexton and Vogel 2002). It is likely that the Type IV system in *Wolbachia* plays a role in the establishment and maintenance of infection and possibly in the generation of reproductive phenotypes.

Genes involved in pathogenicity in bacteria have been found to be frequently associated with regions of anomalous nucleotide composition, possibly owing to transfer from other species or insertion into the genome from plasmids or phage. In the four such regions in *w*Mel (see above; see Table 3), some additional candidates for pathogenicity-related activities are present including a putative penicillin-binding protein (WD0719), genes predicted to be involved in cell wall synthesis (WD0095–WD0098, including D-alanine-D-alanine ligase, a putative FtsQ, and D-alanyl-D-alanine carboxy peptidase) and a multidrug resistance protein (WD0099). In addition, we have identified a cluster of genes in one of the phage regions that may also have some role in host–symbiont interactions. This cluster (WD0611–WD0621) is embedded within the WO-B phage region of the genome (see Figure 2) and contains many genes that encode proteins with putative roles in the synthesis and degradation of surface polysaccharides, including a UDP-glucose 6-dehydrogenase (WD0620). Since this cluster appears to be normal in terms of phylogeny relative to other genes in the genome (i.e., the genes in this region have normal *w*Mel nucleotide composition and branch in phylogenetic trees with genes from other α-Proteobacteria), it is not likely to have been acquired from other species. However, it is possible that these genes can be transferred among *Wolbachia* strains via the phage, which in turn could lead to some variation in host–symbiont interactions between *Wolbachia* strains.

Of particular interest for host-interaction functions are the large number of genes that encode proteins that contain ankyrin repeats (Table 4). Ankyrin repeats, a tandem motif of around 33 amino acids, are found mainly in eukaryotic proteins, where they are known to mediate protein–protein interactions (Caturegli et al. 2000). While they have been found in bacteria before, they are usually present in only a few copies per species. *w*Mel has 23 ankyrin repeat-containing genes, the most currently described for a prokaryote, with C. burnetti being next with 13. This is particularly striking given *w*Mel's relatively small genome size. The functions of the ankyrin repeat-containing proteins in *w*Mel are difficult to predict since most have no sequence similarity outside the ankyrin domains to any proteins of known function. Many lines of evidence suggest that the *w*Mel ankyrin domain proteins are involved in regulating host cell-cycle or cell division or interacting with the host cytoskeleton: (i) many ankyrin-containing proteins in eukaryotes are thought to be involved in linking membrane proteins to the cytoskeleton (Hryniewicz-Jankowska et al. 2002); (ii) an ankyrin-repeat protein of Ehrlichia phagocytophila binds condensed chromatin of host cells and may be involved in host cell-cycle regulation (Caturegli et al. 2000); (iii) some of the proteins that modify the activity of cell-cycle-regulating proteins in D. melanogaster contain ankyrin repeats (Elfring et al. 1997); and (iv) the *Wolbachia* strain that infects the wasp Nasonia vitripennis induces cytoplasmic incompatibility, likely by interacting with these same cell-cycle proteins (Tram and Sullivan 2002). Of the ankyrin-containing proteins in *w*Mel, those worth exploring in more detail include the several that are predicted to be surface targeted or secreted (Table 4) and thus could be targeted to the host nucleus. It is also possible that some of the other ankyrin-containing proteins are secreted via the Type IV secretion system in a targeting signal independent pathway. We call particular attention to three of the ankyrin-containing proteins (WD0285, WD0636, and WD0637), which are among the very few genes, other than those encoding components of the translation apparatus, that have significantly biased codon usage relative to what is expected based on GC content, suggesting they may be highly expressed.

**Table:**
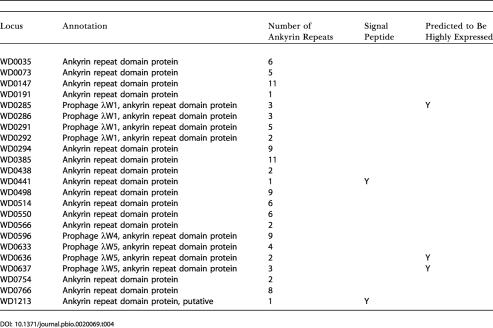
**Table 4.** Ankyrin-Domain Containing Proteins Encoded by the *w*Mel Genome

### Conclusions

Analysis of the *w*Mel genome reveals that it is unique among sequenced genomes of intracellular organisms in that it is both streamlined and massively infected with mobile genetic elements. The persistence of these elements in the genome for apparently long periods of time suggests that *w*Mel is inefficient at getting rid of them, likely a result of experiencing severe population bottlenecks during every cycle of transovarial transmission as well as during sweeps through host populations. Integration of evolutionary reconstructions and genome analysis (phylogenomics) has provided insights into the biology of *Wolbachia*, helped identify genes that likely play roles in the unusual effects *Wolbachia* have on their host, and revealed many new details about the evolution of *Wolbachia* and mitochondria. Perhaps most importantly, future studies of *Wolbachia* will benefit both from this genome sequence and from the ability to study host–symbiont interactions in a host (D. melanogaster) well-suited for experimental studies.

## Materials and Methods

### 

#### Purification/source of DNA

*w*Mel DNA was obtained from *D. melanogaster yw* ^67c23^ flies that naturally carry the *w*Mel infection. *w*Mel was purified from young adult flies on pulsed-field gels as described previously (Sun et al. 2001). Plugs were digested with the restriction enzyme AscI (GG^CGCGCC), which cuts the bacterial chromosome twice (Sun et al. 2001), aiding in the entry of the DNA into agarose gels. After electrophoresis, the resulting two bands were recovered from the gel and stored in 0.5 M EDTA (pH 8.0). DNA was extracted from the gel slices by first washing in TE (Tris–HCl and EDTA) buffer six times for 30 min each to dilute EDTA followed by two 1-h washes in β-agarase buffer (New England Biolabs, Beverly, Massachusetts, United States). Buffer was then removed and the blocks melted at 70°C for 7 min. The molten agarose was cooled to 40°C and then incubated in β-agarase (1 U/100 μl of molten agarose) for 1 h. The digest was cooled to 4°C for 1 h and then centrifuged at 4,100 × *g*_max_ for 30 min at 4°C to remove undigested agarose. The supernatant was concentrated on a Centricon YM-100 microconcentrator (Millipore, Bedford, Massachusetts, United States) after prerinsing with 70% ethanol followed by TE buffer and, after concentration, rinsed with TE. The retentate was incubated with proteinase K at 56°C for 2 h and then stored at 4°C. *w*Mel DNA for gap closure was prepared from approximately 1,000 *Drosophila* adults using the Holmes–Bonner urea/phenol:chloroform protocol (Holmes and Bonner 1973) to prepare total fly DNA.

#### Library construction/sequencing/closure

The complete genome sequence was determined using the whole-genome shotgun method (Venter et al. 1996). For the random shotgun-sequencing phase, libraries of average size 1.5–2.0 kb and 4.0–8.0 kb were used. After assembly using the TIGR Assembler (Sutton et al. 1995), there were 78 contigs greater than 5000 bp, 186 contigs greater than 3000 bp, and 373 contigs greater than 1500 bp. This number of contigs was unusually high for a 1.27 Mb genome. An initial screen using BLASTN searches against the nonredundant database in GenBank and the Berkeley *Drosophila* Genome Project site (http://www.fruitfly.org/blast/) showed that 3,912 of the 10,642 contigs were likely contaminants from the *Drosophila* genome. To aid in closure, the assemblies were rerun with all sequences of likely host origin excluded. Closure, which was made very difficult by the presence of a large amount of repetitive DNA (see below), was done using a mix of primer walking, generation, and sequencing of transposon-tagged libraries of large insert clones and multiplex PCR (Tettelin et al. 1999). The final sequence showed little evidence for polymorphism within the population of *Wolbachia* DNA. In addition, to obtain sequence across the AscI-cut sites, PCR was performed on undigested DNA. It is important to point out that the reason significant host contamination does not significantly affect symbiont genome assembly is that most of the *Drosophila* contigs were small due to the approximately 100-fold difference in genome sizes between host (approximately 180 Mb) and *w*Mel (1.2 Mb).

Since it has been suggested that *Wolbachia* and their hosts may undergo lateral gene transfer events (Kondo et al. 2002), genome assemblies were rerun using all of the shotgun and closure reads without excluding any sequences that appeared to be of host origin. Only five assemblies were found to match both the D. melanogaster genome and the *w*Mel assembly. Primers were designed to match these assemblies and PCR attempted from total DNA of *w*Mel infected D. melanogaster. In each case, PCR was unsuccessful, and we therefore presume that these assemblies are the result of chimeric cloning artifacts. The complete sequence has been given GenBank accession ID AE017196 and is available at http://www.tigr.org/tdb.

#### Repeats

Repeats were identified using RepeatFinder (Volfovsky et al. 2001), which makes use of the REPuter algorithm (Kurtz and Schleiermacher 1999) to find maximal-length repeats. Some manual curation and BLASTN and BLASTX searches were used to divide repeat families into different classes.

#### Annotation

Identification of putative protein-encoding genes and annotation of the genome was done as described previously (Eisen et al. 2002). An initial set of ORFs likely to encode proteins (CDS) was identified with GLIMMER (Salzberg et al. 1998). Putative proteins encoded by the CDS were examined to identify frameshifts or premature stop codons compared to other species. The sequence traces for each were reexamined and, for some, new sequences were generated. Those for which the frameshift or premature stops were of high quality were annotated as “authentic” mutations. Functional assignment, identification of membrane-spanning domains, determination of paralogous gene families, and identification of regions of unusual nucleotide composition were performed as described previously (Tettelin et al. 2001). Phylogenomic analysis (Eisen 1998a; Eisen and Fraser 2003) was used to aid in functional predictions. Alignments and phylogenetic trees were generated as described (Salzberg et al. 2001).

#### Comparative genomics

All putative *w*Mel proteins were searched using BLASTP against the predicted proteomes of published complete organismal genomes and a set of complete plastid, mitochondrial, plasmid, and viral genomes. The results of these searches were used (i) to analyze the phylogenetic profile (Pellegrini et al. 1999; Eisen and Wu 2002), (ii) to identify putative lineage-specific duplications (those proteins with a top *E*-value score to another protein from *w*Mel), and (iii) to determine the presence of homologs in different species. Orthologs between the *w*Mel genome and that of the two *Rickettsia* species were identified by requiring mutual best-hit relationships among all possible pairwise BLASTP comparisons, with some manual correction. Those genes present in both *Rickettsia* genomes as well as other bacterial species, but not *w*Mel, were considered to have been lost in the *w*Mel branch (see Table S3). Genes present in only one or two of the three species were considered candidates for gene loss or lateral transfer and were also used to identify possible biological differences between these species (see Table S3). For the *w*Mel genes not in the *Rickettsia* genomes, proteins were searched with BLASTP against the TIGR NRAA database. Protein sequences of their homologs were aligned with CLUSTALW and manually curated. Neighbor-joining trees were constructed using the PHYLIP package.

#### Phylogenetic analysis of mitochondrial proteins

For phylogenetic analysis, the set of all 38 proteins encoded in both the Marchantia polymorpha and Reclinomonas americana (Lang et al. 1997) mitochondrial genomes were collected. Acanthamoeba castellanii was excluded due to high divergence and extremely long evolutionary branches. Six genes were excluded from further analysis because they were too poorly conserved for alignment and phylogenetic analysis (*nad7*, *rps10*, *sdh3*, *sdh4*, *tatC*, and *yejV*), leaving 32 genes for investigation: *atp6*, *atp9*, *atpA*, *cob*, *cox1*, *cox2*, *cox3*, *nad1*, *nad2*, *nad3*, *nad4*, *nad4L*, *nad5*, *nad6*, *nad9*, *rpl16*, *rpl2*, *rpl5*, *rpl6*, *rps1*, *rps11*, *rps12*, *rps13*, *rps14*, *rps19*, *rps2*, *rps3*, *rps4*, *rps7*, *rps8*, *yejR*, and *yejU*. Using FASTA with the mitochondrial proteins as a query, homologs were identified from the genomes of seven α-Proteobacteria: two intracellular symbionts (*W. pipientis w*Mel and Rickettsia prowazekii) and five free-living forms (Sinorhozobium loti, Agrobacterium tumefaciens, Brucella melitensis, Mesorhizobium loti, and *Rhodopseudomonas* sp.). Escherichia coli and Neisseria meningitidis were used as outgroups. Caulobacter crescentus was excluded from analysis because homologs of some of the 32 genes were not found in the current annotation. In the event that more than one homolog was identified per genome, the one with the greatest sequence identity to the mitochondrial query was retrieved. Proteins were aligned using CLUSTALW (Thompson et al. 1994) and concatenated. To reduce the influence of poorly aligned regions, all sites that contained a gap at any position were excluded from analysis, leaving 6,776 positions per genome for analysis. The data contained extreme amino acid bias: all sequences failed the χ^2^ test at *p* = 0.95 for deviation from amino acid frequency distribution assumed under either the JTT or mtREV24 models as determined with PUZZLE (Strimmer and von Haeseler 1996). When the data were iteratively purged of highly variable sites using the method described (Hansmann and Martin 2000), amino acid composition gradually came into better agreement with acid frequency distribution assumed by the model. The longest dataset in which all sequences passed the χ^2^ test at *p* = 0.95 consisted of the 3,100 least polymorphic sites. PROTML (Adachi and Hasegawa 1996) analyses of the 3,100-site data using the JTT model detected mitochondria as sisters of the five free-living α-Proteobacteria with low (72%) support, whereas PUZZLE, using the same data, detected mitochondria as sisters of the two intracellular symbionts, also with low (85%) support. This suggested the presence of conflicting signal in the less-biased subset of the data. Therefore, protein log determinants (LogDet) were used to infer distances from the 6,776-site data, since the method can correct for amino acid bias (Lockhart et al. 1994), and Neighbor-Net (Bryant and Moulton 2003) was used to display the resulting matrix, because it can detect and display conflicting signal. The result (see Figure 5A) shows both signals. In no analysis was a sister relationship between *Rickettsia* and mitochondria detected.

For analyses of individual genes, the 63 proteins encoded in the *Reclinomonas* mitochondrial genome were compared with FASTA to the proteins from 49 sequenced eubacterial genomes, which included the α-Proteobacteria shown in Figure 5, R. conorii, and *Magnetococcus* MC1, one of the more divergent α-Proteobacteria. Of those proteins, 50 had sufficiently well-conserved homologs to perform phylogenetic analyses. Homologs were aligned and subjected to phylogenetic analysis with PROTML (Adachi and Hasegawa 1996).

#### Analysis of *wspB* sequences

To compare *wspB* sequences from different *Wolbachia* strains, PCR was done on total DNA extracted from the following sources: *w*Ri was obtained from infected adult D. simulans, Riverside strain; *w*AlbB was obtained from the infected Aa23 cell line (O'Neill et al. 1997b), and *D. immitis Wolbachia* was extracted from adult worm tissue. DNA extraction and PCR were done as previously described (Zhou et al. 1998) with *wspB*-specific primers (*wspB*-F, 5′-TTTGCAAGTGAAACAGAAGG and *wspB*-R, 5′-GCTTTGCTGGCAAAATGG). PCR products were cloned into pGem-T vector (Promega, Madison, Wisconsin, United States) as previously described (Zhou et al. 1998) and sequenced (Genbank accession numbers AJ580921–AJ508923). These sequences were compared to previously sequenced *wsp* genes for the same *Wolbachia* strains (Genbank accession numbers AF020070, AF020059, and AJ252062). The four partial *wsp* sequences were aligned using CLUSTALV (Higgins et al. 1992) based on the amino acid translation of each gene and similarly with the *wspB* sequences. Genetic distances were calculated using the Kimura 2 parameter method and are reported in Table S5.

#### Type IV secretion system

To determine whether the *vir*-like CDSs, as well as adjacent ORFs, were actively expressed within *w*Mel as two polycistronic operons, RT-PCR was used. Total RNA was isolated from infected *D. melanogaster yw* ^67c23^ adults using Trizol reagent (Invitrogen, Carlsbad, California, United States) and cDNA synthesized using SuperScript III RT (Invitrogen) using primers *wspB*R, WD0817R, WD0853R, and WD0852R. RNA isolation and RT were done according to manufacturer's protocols, with the exception that suggested initial incubation of RNA template and primers at 65°C for 5 min and final heat denaturation of RT-enzyme at 70°C for 15 min were not done. PCR was done using r*Taq* (Takara, Kyoto, Japan), and several primer sets were used to amplify regions spanning adjacent CDSs for most of the two operons. For operon virB3-WD0853, the following primers were used: (*virB3*-*virB4*)F, (*virB3*-*virB4*)R, (*virB6*-WD0856)F, (*virB6*-WD0856)R, (WD0856-WD0855)F, (WD0856-WD0855)R, (WD0854-WD0853)F, (WD0854-WD0853)R. For operon *virB8*-*wspB*, the following primers were used: (*virB8*-*virB9*)F, (*virB8*-*virB9*)R, (*virB9*-*virB11*)F, (*virB9*-*virB11*)R, (*virB11*-*virD4*)F, (*virB11*-*virD4*)R, (*virD4*-*wspB*)F, and (*virD4*-*wspB*)R. The coexpression of *virB4* and *virB6*, as well as WD0855 and WD0854, was confirmed within the putative *virB3*-WD0853 operon using nested PCR with the following primers: (*virB4*-*virB6*)F1, (*virB4*-*virB6*)R1, (*virB4*-*virB6*)F2, (*virB4*-*virB6*)R2, (WD0855-WD0854)F1, (WD0855-WD0854)R1, (WD0855-WD0854)F2, and (WD0855-WD0854)R2. All ORFs within the putative *virB8*-*wspB* operon were shown to be coexpressed and are thus considered to be a genuine operon. All products were amplified only from RT-positive reactions (see Figure 6). Primer sequences are given in Table S9.

## Supporting Information

Figure S1 Phage Trees Phylogenetic tree showing the relationship between WO-A and WO-B phage from *w*Mel with reported phage from *w*Kue and *w*Tai. The tree was generated from a CLUSTALW multiple sequence alignment (Thompson et al. 1994) using the PROTDIST and NEIGHBOR programs of PHYLIP (Felsenstein 1989).
(60 KB PDF).

Figure S2 Plot of the Effective Number of Codons against GC Content at the Third Codon Position (GC3) Proteins with fewer than 100 residues are excluded from this analysis because their effective number of codon (ENc) values are unreliable. The curve shows the expected ENc values if codon usage bias is caused by GC variation alone. Colors: yellow, hypothetical; purple, mobile element; blue, others. Most of the variation in codon bias can be traced to variation in GC, indicating that the mutation forces dominate the *w*Mel codon usage. Multivariate analysis of codon usage was performed using the CODONW package (available from http://www.molbiol.ox.ac.uk/cu/codonW.html).
(289 KB PDF).

Figure S3 Predicted Metabolism and Transport in *w*Mel Overview of the predicted metabolism (energy production and organic compounds) and transport in *w*Mel*.* Transporters are grouped by predicted substrate specificity: inorganic cations (green), inorganic anions (pink), carbohydrates (yellow), and amino acids/peptides/amines/purines and pyrimidines (red). Transporters in the drug-efflux family (labeled as “drugs”) and those of unknown specificity are colored black. Arrows indicate the direction of transport. Energy-coupling mechanisms are also shown: solutes transported by channel proteins (double-headed arrow); secondary transporters (two-arrowed lines, indicating both the solute and the coupling ion); ATP-driven transporters (ATP hydrolysis reaction); unknown energy-coupling mechanism (single arrow). Transporter predictions are based upon a phylogenetic classification of transporter proteins (Paulsen et al. 1998).
(167 KB PDF).

Table S1 Repeats of Greater Than 50 bp in the *w*Mel Genome (with Coordinates) (649 KB DOC).

Table S2 Inactivated Genes in the *w*Mel Genome (147 KB DOC).

Table S3 Ortholog Comparison with *Rickettsia* spp (718 KB XLS).

Table S4 Putative Lineage-Specific Gene Duplications in *w*Mel (116 KB DOC).

Table S5 Genetic Distances as Calculated for Alignments of *wsp* and *wspB* Gene Sequences from the Same *Wolbachia* Strains (24 KB DOC).

Table S6 Putative DNA Repair and Recombination Genes in the *w*Mel Genome (26 KB DOC).

Table S7 Phylogenetic Results for Concatenated Data of 32 Mitochondrial Proteins (34 KB DOC).

Table S8 Individual Phylogenetic Results for *Reclinomonas* Mitochondrial DNA-Encoded Proteins (117 KB DOC).

Table S9 PCR Primers (47 KB DOC).

### Accession Numbers

The complete sequence for *w*Mel has been given GenBank (http://www.ncbi.nlm.nih.gov/Genbank/) accession ID number AE017196 and is available through the TIGR Comprehensive Microbial Resourceat http://www.tigr.org/tigr-scripts/CMR2/GenomePage3.spl?database=dmg

The GenBank accession numbers for other sequences discussed in this paper are AF020059 (*Wolbachia* sp. *w*AlbB outer surface protein precursor *wsp* gene), AF020070 (*Wolbachia* sp. *w*Ri outer surface protein precursor *wsp* gene), AJ252062 (*Wolbachia* endosymbiont of D. immitis sp. gene for surface protein), AJ580921 (*Wolbachia* endosymbiont of D. immitis partial *wspB* gene for *Wolbachia* surface protein B), AJ580922 (*Wolbachia* endosymbiont of A. albopictus partial *wspB* gene for *Wolbachia* surface protein B), and AJ580923 (*Wolbachia* endosymbiont of D. simulans partial *wspB* gene for *Wolbachia* surface protein B).

## Abbreviations

CDS: coding sequence
ENc: effective number of codons
IS: insertion sequence
LPS: lipopolysaccharide
RT: reverse transcription
TIGR: The Institute for Genomic Research
