# Supplementary figures and images for: Phylogenomics of the Reproductive Parasite Wolbachia pipientis wMel: A Streamlined Genome Overrun by Mobile Genetic Elements
Source: PLoS Biol. 2004 Mar 16;2(3):e69. doi: 10.1371/journal.pbio.0020069 (PMC368164; doi:10.1371/journal.pbio.0020069)

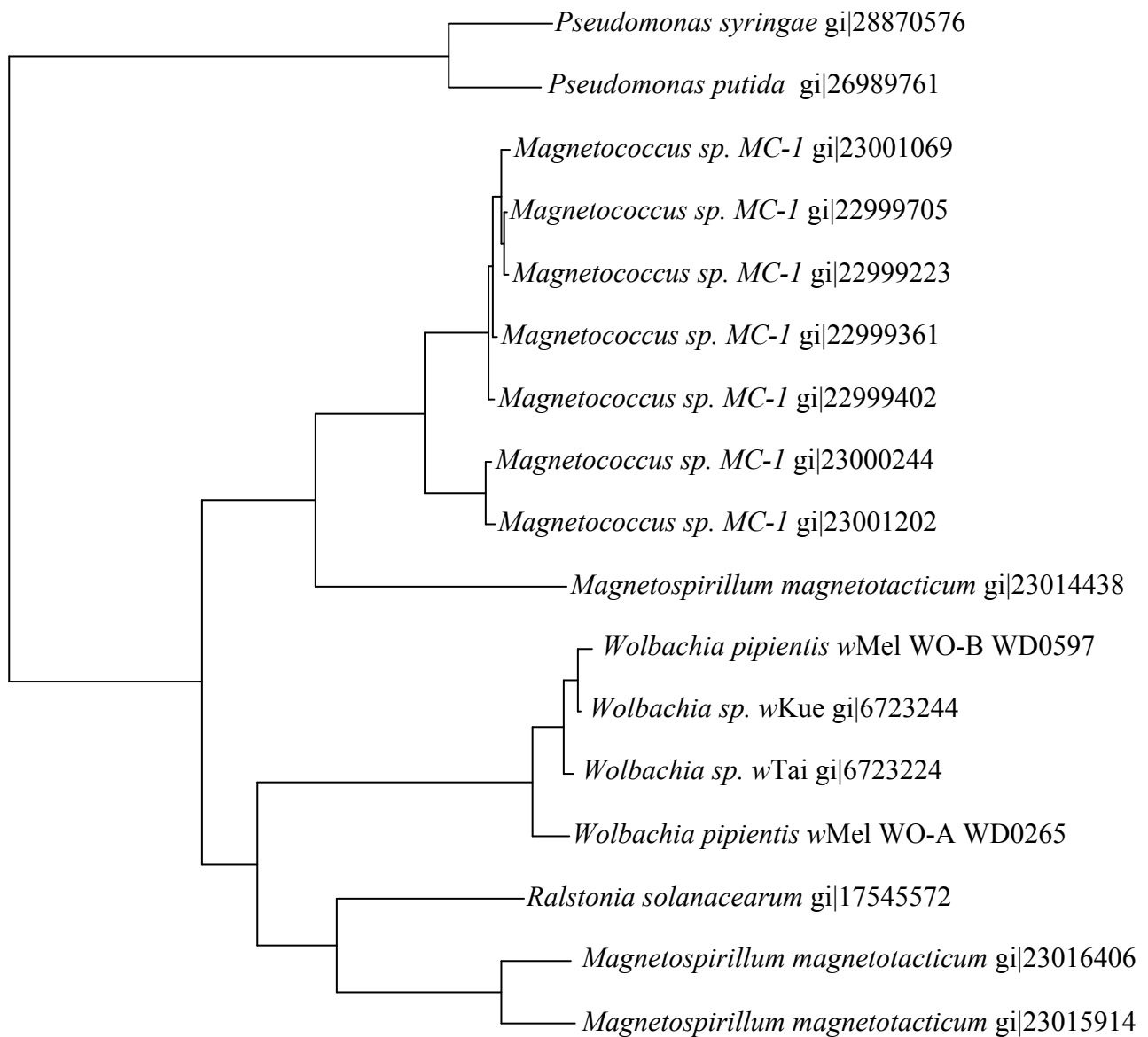

Supplement: Figure S1 — Phylogenetic tree showing the relationship between WO-A and WO-B phage from wMel with reported phage from wKue and wTai. The tree was generated from a CLUSTALW multiple sequence alignment (Thompson et al. 1994) using the PROTDIST and NEIGHBOR programs of PHYLIP (Felsenstein 1989). (60 KB PDF). [file pbio.0020069.sg001.pdf]

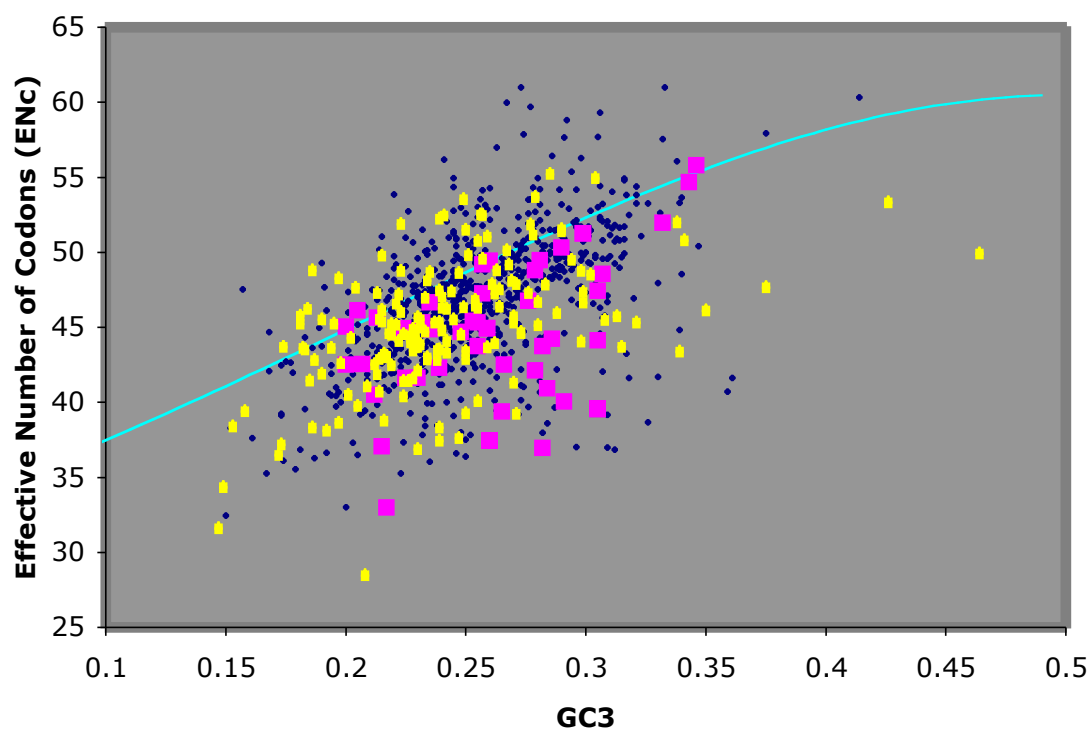

Supplement: Figure S2 — Proteins with fewer than 100 residues are excluded from this analysis because their effective number of codon (ENc) values are unreliable. The curve shows the expected ENc values if codon usage bias is caused by GC variation alone. Colors: yellow, hypothetical; purple, mobile element; blue, others. Most of the variation in codon bias can be traced to variation in GC, indicating that the mutation forces dominate the wMel codon usage. Multivariate analysis of codon usage was performed using the CODONW package (available from http://www.molbiol.ox.ac.uk/cu/codonW.html). (289 KB PDF). [file pbio.0020069.sg002.pdf]

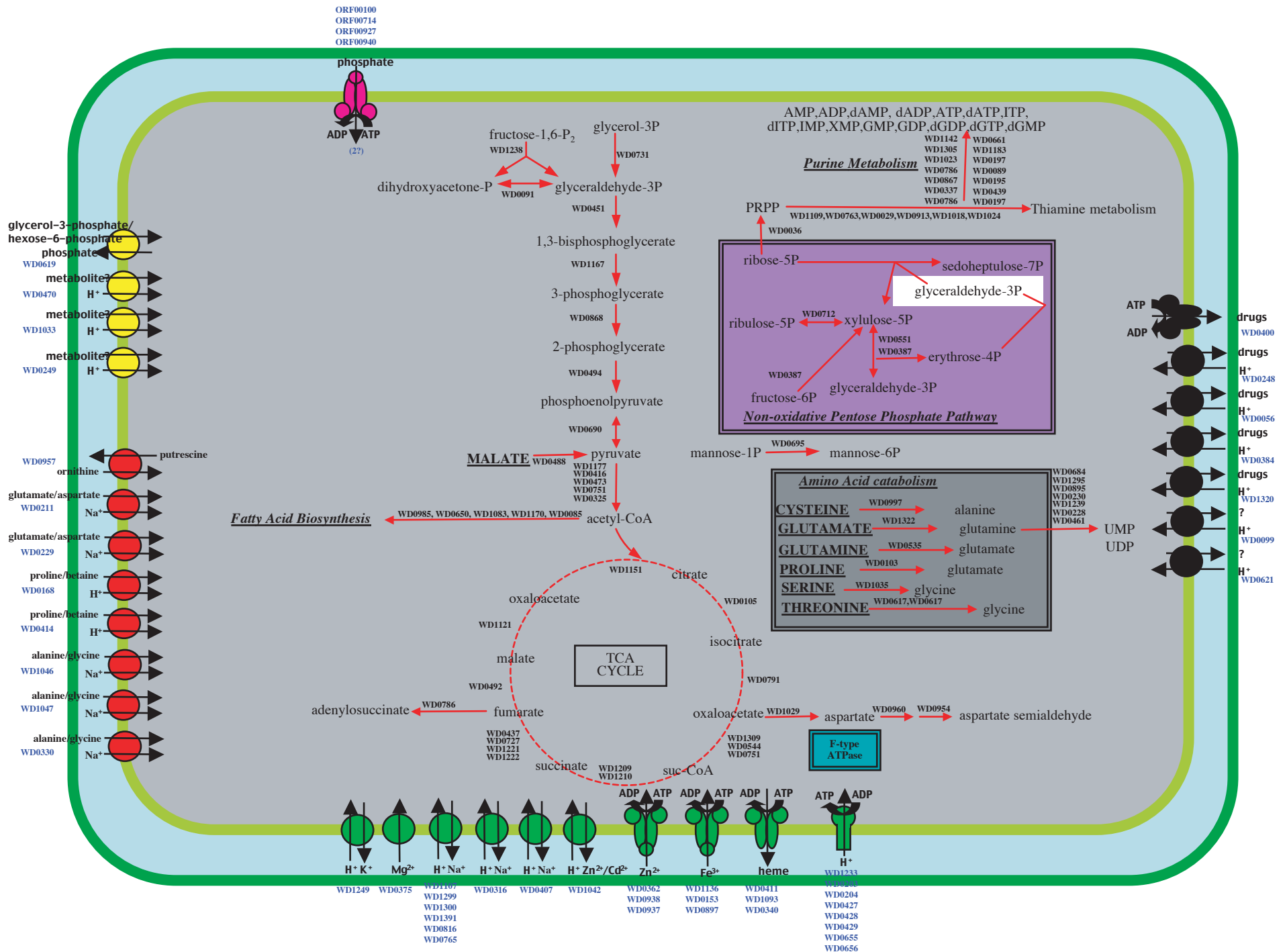

Supplement: Figure S3 — Overview of the predicted metabolism (energy production and organic compounds) and transport in wMel. Transporters are grouped by predicted substrate specificity: inorganic cations (green), inorganic anions (pink), carbohydrates (yellow), and amino acids/peptides/amines/purines and pyrimidines (red). Transporters in the drug-efflux family (labeled as “drugs”) and those of unknown specificity are colored black. Arrows indicate the direction of transport. Energy-coupling mechanisms are also shown: solutes transported by channel proteins (double-headed arrow); secondary transporters (two-arrowed lines, indicating both the solute and the coupling ion); ATP-driven transporters (ATP hydrolysis reaction); unknown energy-coupling mechanism (single arrow). Transporter predictions are based upon a phylogenetic classification of transporter proteins (Paulsen et al. 1998). (167 KB PDF). [file pbio.0020069.sg003.pdf]
