# Supplementary material for: Phylogenomics of the Reproductive Parasite Wolbachia pipientis wMel: A Streamlined Genome Overrun by Mobile Genetic Elements
Source: PLoS Biol. 2004 Mar 16;2(3):e69. doi: 10.1371/journal.pbio.0020069 (PMC368164; doi:10.1371/journal.pbio.0020069)
Supplement: Table S1 — (649 KB DOC). [file pbio.0020069.st001.doc]

Table S1. Repeats of greater than 50 bp in the *w*Mel genome (with coordinates). These were identified using the RepeatFinder program as described in the text.

| Repeat Class | Repeat Name | Start | End | Length |
| --- | --- | --- | --- | --- |
| 1 | C1R1 | 12456 | 12516 | 61 |
| 1 | C1R2 | 1166460 | 1166520 | 61 |
| 2 | C2R1 | 12560 | 12631 | 72 |
| 2 | C2R2 | 778735 | 778817 | 83 |
| 2 | C2R3 | 943659 | 943577 | 83 |
| 3 | C3R1 | 12731 | 12800 | 70 |
| 3 | C3R2 | 12735 | 12810 | 76 |
| 3 | C3R3 | 12755 | 12844 | 90 |
| 3 | C3R4 | 123637 | 123568 | 70 |
| 3 | C3R5 | 997684 | 997609 | 76 |
| 3 | C3R6 | 1190334 | 1190245 | 90 |
| 3 | C3R7 | 1230663 | 1230601 | 63 |
| 4 | C4R1 | 12925 | 12870 | 56 |
| 4 | C4R2 | 52007 | 51894 | 114 |
| 4 | C4R3 | 290275 | 290484 | 210 |
| 4 | C4R4 | 339153 | 339362 | 210 |
| 4 | C4R5 | 997438 | 997490 | 53 |
| 4 | C4R6 | 1190018 | 1190122 | 105 |
| 4 | C4R7 | 1190141 | 1190200 | 60 |
| 4 | C4R8 | 1230318 | 1230441 | 124 |
| 4 | C4R9 | 1230438 | 1230494 | 57 |
| 5 | C5R1 | 12968 | 12909 | 60 |
| 5 | C5R2 | 997450 | 997509 | 60 |
| 6 | C6R1 | 12990 | 13040 | 51 |
| 6 | C6R2 | 997428 | 997378 | 51 |
| 7 | C7R1 | 13116 | 13174 | 59 |
| 7 | C7R2 | 462013 | 461955 | 59 |
| 8 | C8R1 | 16526 | 16327 | 200 |
| 8 | C8R2 | 658904 | 659103 | 200 |
| 9 | C9R1 | 17443 | 16528 | 916 |
| 9 | C9R2 | 657987 | 658902 | 916 |
| 10 | C10R1 | 34087 | 34140 | 54 |
| 10 | C10R2 | 293819 | 293766 | 54 |
| 11 | C11R1 | 34500 | 34108 | 393 |
| 11 | C11R10 | 123579 | 123529 | 51 |
| 11 | C11R11 | 151298 | 151535 | 238 |
| 11 | C11R12 | 151391 | 151693 | 303 |
| 11 | C11R13 | 151618 | 151728 | 111 |
| 11 | C11R14 | 171054 | 171291 | 238 |
| 11 | C11R15 | 171293 | 171493 | 201 |
| 11 | C11R16 | 178433 | 178506 | 74 |
| 11 | C11R17 | 178443 | 178519 | 77 |
| 11 | C11R18 | 178598 | 178671 | 74 |
| 11 | C11R19 | 380088 | 380580 | 493 |
| 11 | C11R2 | 34565 | 34491 | 75 |
| 11 | C11R20 | 380505 | 380615 | 111 |
| 11 | C11R21 | 417877 | 417713 | 165 |
| 11 | C11R22 | 417917 | 417792 | 126 |
| 11 | C11R23 | 418021 | 417919 | 103 |
| 11 | C11R24 | 418084 | 417999 | 86 |
| 11 | C11R25 | 418111 | 418034 | 78 |
| 11 | C11R26 | 432761 | 432815 | 55 |
| 11 | C11R27 | 432813 | 432868 | 56 |
| 11 | C11R28 | 432870 | 432990 | 121 |
| 11 | C11R29 | 432905 | 433067 | 163 |
| 11 | C11R3 | 34577 | 34504 | 74 |
| 11 | C11R30 | 432992 | 433090 | 99 |
| 11 | C11R31 | 462352 | 462039 | 314 |
| 11 | C11R32 | 462419 | 462364 | 56 |
| 11 | C11R33 | 462445 | 462369 | 77 |
| 11 | C11R34 | 693498 | 693801 | 304 |
| 11 | C11R35 | 693521 | 693950 | 430 |
| 11 | C11R36 | 693681 | 693986 | 306 |
| 11 | C11R37 | 714146 | 713868 | 279 |
| 11 | C11R38 | 714301 | 713998 | 304 |
| 11 | C11R39 | 752680 | 752896 | 217 |
| 11 | C11R4 | 54562 | 54497 | 66 |
| 11 | C11R40 | 752898 | 753111 | 214 |
| 11 | C11R41 | 776933 | 777006 | 74 |
| 11 | C11R42 | 776940 | 777019 | 80 |
| 11 | C11R43 | 792079 | 791658 | 422 |
| 11 | C11R44 | 792170 | 792096 | 75 |
| 11 | C11R45 | 811821 | 811877 | 57 |
| 11 | C11R46 | 811911 | 812002 | 92 |
| 11 | C11R47 | 812053 | 812108 | 56 |
| 11 | C11R48 | 812057 | 812322 | 266 |
| 11 | C11R49 | 832635 | 832314 | 322 |
| 11 | C11R5 | 67629 | 67237 | 393 |
| 11 | C11R50 | 832839 | 832333 | 507 |
| 11 | C11R51 | 832855 | 832706 | 150 |
| 11 | C11R52 | 839912 | 839988 | 77 |
| 11 | C11R53 | 839938 | 840036 | 99 |
| 11 | C11R54 | 840038 | 840309 | 272 |
| 11 | C11R55 | 848562 | 849071 | 510 |
| 11 | C11R56 | 848801 | 849086 | 286 |
| 11 | C11R57 | 848893 | 849106 | 214 |
| 11 | C11R58 | 854406 | 854286 | 121 |
| 11 | C11R59 | 854823 | 854329 | 495 |
| 11 | C11R6 | 67653 | 67574 | 80 |
| 11 | C11R60 | 943095 | 942997 | 99 |
| 11 | C11R61 | 943182 | 943005 | 178 |
| 11 | C11R62 | 943222 | 943097 | 126 |
| 11 | C11R63 | 943326 | 943184 | 143 |
| 11 | C11R64 | 943433 | 943328 | 106 |
| 11 | C11R65 | 943454 | 943380 | 75 |
| 11 | C11R66 | 1080790 | 1080494 | 297 |
| 11 | C11R67 | 1080815 | 1080536 | 280 |
| 11 | C11R68 | 1080842 | 1080740 | 103 |
| 11 | C11R69 | 1080894 | 1080820 | 75 |
| 11 | C11R7 | 67673 | 67620 | 54 |
| 11 | C11R70 | 1080929 | 1080850 | 80 |
| 11 | C11R71 | 1130293 | 1130404 | 112 |
| 11 | C11R72 | 1130319 | 1130585 | 267 |
| 11 | C11R73 | 1130587 | 1130704 | 118 |
| 11 | C11R74 | 1166661 | 1166756 | 96 |
| 11 | C11R75 | 1166706 | 1166791 | 86 |
| 11 | C11R76 | 1166758 | 1166842 | 85 |
| 11 | C11R77 | 1166842 | 1167126 | 285 |
| 11 | C11R78 | 1216670 | 1216516 | 155 |
| 11 | C11R79 | 1216820 | 1216542 | 279 |
| 11 | C11R8 | 90084 | 90147 | 64 |
| 11 | C11R80 | 1216907 | 1216809 | 99 |
| 11 | C11R81 | 1216932 | 1216822 | 111 |
| 11 | C11R82 | 1216952 | 1216846 | 107 |
| 11 | C11R83 | 1217060 | 1216911 | 150 |
| 11 | C11R84 | 1230794 | 1230742 | 53 |
| 11 | C11R85 | 1248067 | 1247990 | 78 |
| 11 | C11R86 | 1248083 | 1248018 | 66 |
| 11 | C11R87 | 1257034 | 1257085 | 52 |
| 11 | C11R88 | 1257156 | 1257384 | 229 |
| 11 | C11R89 | 1257386 | 1257565 | 180 |
| 11 | C11R9 | 123506 | 123274 | 233 |
| 12 | C12R1 | 1231492 | 1231550 | 59 |
| 12 | C12R2 | 1231827 | 1231885 | 59 |
| 13 | C13R1 | 34638 | 34589 | 50 |
| 13 | C13R2 | 541021 | 541070 | 50 |
| 14 | C14R1 | 35359 | 35450 | 92 |
| 14 | C14R2 | 35475 | 35559 | 85 |
| 14 | C14R3 | 35811 | 35876 | 66 |
| 14 | C14R4 | 36912 | 36996 | 85 |
| 14 | C14R5 | 36940 | 37031 | 92 |
| 15 | C15R1 | 35770 | 35822 | 53 |
| 15 | C15R2 | 36058 | 36110 | 53 |
| 16 | C16R1 | 36223 | 36277 | 55 |
| 16 | C16R2 | 36508 | 36562 | 55 |
| 17 | C17R1 | 37528 | 37474 | 55 |
| 17 | C17R2 | 970615 | 970669 | 55 |
| 18 | C18R1 | 1231018 | 1231070 | 53 |
| 18 | C18R2 | 1231813 | 1231865 | 53 |
| 19 | C19R1 | 37490 | 37547 | 58 |
| 19 | C19R2 | 54564 | 54621 | 58 |
| 19 | C19R3 | 168547 | 168601 | 55 |
| 19 | C19R4 | 521981 | 522402 | 422 |
| 19 | C19R5 | 547418 | 547482 | 65 |
| 19 | C19R6 | 885735 | 885790 | 56 |
| 19 | C19R7 | 885738 | 885795 | 58 |
| 19 | C19R8 | 950462 | 950883 | 422 |
| 19 | C19R9 | 1091783 | 1091701 | 83 |
| 20 | C20R1 | 46026 | 45922 | 105 |
| 20 | C20R2 | 165097 | 164993 | 105 |
| 20 | C20R3 | 165148 | 165031 | 118 |
| 20 | C20R4 | 953576 | 953693 | 118 |
| 21 | C21R1 | 46084 | 46159 | 76 |
| 21 | C21R2 | 165155 | 165230 | 76 |
| 22 | C22R1 | 46397 | 46475 | 79 |
| 22 | C22R2 | 165468 | 165546 | 79 |
| 23 | C23R1 | 46477 | 46580 | 104 |
| 23 | C23R2 | 165548 | 165651 | 104 |
| 24 | C24R1 | 46828 | 47751 | 924 |
| 24 | C24R10 | 873688 | 874612 | 925 |
| 24 | C24R11 | 886195 | 887116 | 922 |
| 24 | C24R12 | 897489 | 896566 | 924 |
| 24 | C24R13 | 1172757 | 1172253 | 505 |
| 24 | C24R14 | 1173175 | 1172759 | 417 |
| 24 | C24R2 | 127152 | 126227 | 926 |
| 24 | C24R3 | 197397 | 198323 | 927 |
| 24 | C24R4 | 308698 | 309624 | 927 |
| 24 | C24R5 | 438674 | 437751 | 924 |
| 24 | C24R6 | 507925 | 508848 | 924 |
| 24 | C24R7 | 533176 | 532253 | 924 |
| 24 | C24R8 | 568215 | 569136 | 922 |
| 24 | C24R9 | 635588 | 634663 | 926 |
| 25 | C25R1 | 49566 | 49290 | 277 |
| 25 | C25R10 | 1089273 | 1089161 | 113 |
| 25 | C25R11 | 1091466 | 1089275 | 2192 |
| 25 | C25R2 | 210581 | 210453 | 129 |
| 25 | C25R3 | 379499 | 376721 | 2779 |
| 25 | C25R4 | 522491 | 525272 | 2782 |
| 25 | C25R5 | 668804 | 671156 | 2353 |
| 25 | C25R6 | 671158 | 671221 | 64 |
| 25 | C25R7 | 950889 | 953241 | 2353 |
| 25 | C25R8 | 952899 | 953277 | 379 |
| 25 | C25R9 | 953299 | 953575 | 277 |
| 26 | C26R1 | 51080 | 51162 | 83 |
| 26 | C26R2 | 865358 | 865276 | 83 |
| 27 | C27R1 | 51284 | 51187 | 98 |
| 27 | C27R2 | 865154 | 865251 | 98 |
| 28 | C28R1 | 51286 | 51504 | 219 |
| 28 | C28R2 | 51303 | 51672 | 370 |
| 28 | C28R3 | 51559 | 51726 | 168 |
| 28 | C28R4 | 864879 | 864712 | 168 |
| 28 | C28R5 | 865152 | 864934 | 219 |
| 28 | C28R6 | 872745 | 872376 | 370 |
| 29 | C29R1 | 51728 | 51864 | 137 |
| 29 | C29R10 | 178670 | 178783 | 114 |
| 29 | C29R11 | 178742 | 178924 | 183 |
| 29 | C29R12 | 178806 | 179031 | 226 |
| 29 | C29R13 | 293384 | 293484 | 101 |
| 29 | C29R14 | 293483 | 293592 | 110 |
| 29 | C29R15 | 293684 | 293791 | 108 |
| 29 | C29R16 | 293712 | 293810 | 99 |
| 29 | C29R17 | 331481 | 331373 | 109 |
| 29 | C29R18 | 331498 | 331439 | 60 |
| 29 | C29R19 | 331502 | 331446 | 57 |
| 29 | C29R2 | 90184 | 90284 | 101 |
| 29 | C29R20 | 331595 | 331520 | 76 |
| 29 | C29R21 | 331636 | 331543 | 94 |
| 29 | C29R22 | 331667 | 331555 | 113 |
| 29 | C29R23 | 331778 | 331597 | 182 |
| 29 | C29R24 | 331798 | 331682 | 117 |
| 29 | C29R25 | 388176 | 388289 | 114 |
| 29 | C29R26 | 388291 | 388516 | 226 |
| 29 | C29R27 | 388432 | 388532 | 101 |
| 29 | C29R28 | 458157 | 458386 | 230 |
| 29 | C29R29 | 458290 | 458417 | 128 |
| 29 | C29R3 | 90203 | 90392 | 190 |
| 29 | C29R30 | 458329 | 458435 | 107 |
| 29 | C29R31 | 458378 | 458456 | 79 |
| 29 | C29R32 | 731009 | 731096 | 88 |
| 29 | C29R33 | 731114 | 731223 | 110 |
| 29 | C29R34 | 731152 | 731295 | 144 |
| 29 | C29R35 | 731291 | 731410 | 120 |
| 29 | C29R36 | 864710 | 864574 | 137 |
| 29 | C29R37 | 980974 | 981161 | 188 |
| 29 | C29R38 | 981109 | 981200 | 92 |
| 29 | C29R39 | 981273 | 981380 | 108 |
| 29 | C29R4 | 90333 | 90439 | 107 |
| 29 | C29R40 | 1035508 | 1035402 | 107 |
| 29 | C29R41 | 1035701 | 1035510 | 192 |
| 29 | C29R42 | 1035744 | 1035562 | 183 |
| 29 | C29R43 | 1035815 | 1035746 | 70 |
| 29 | C29R44 | 1067955 | 1067796 | 160 |
| 29 | C29R45 | 1068008 | 1067921 | 88 |
| 29 | C29R46 | 1068031 | 1067957 | 75 |
| 29 | C29R47 | 1068144 | 1068054 | 91 |
| 29 | C29R48 | 1094010 | 1094063 | 54 |
| 29 | C29R49 | 1094131 | 1094198 | 68 |
| 29 | C29R5 | 90341 | 90484 | 144 |
| 29 | C29R50 | 1094297 | 1094353 | 57 |
| 29 | C29R51 | 1094355 | 1094426 | 72 |
| 29 | C29R52 | 1094361 | 1094435 | 75 |
| 29 | C29R53 | 1115009 | 1115100 | 92 |
| 29 | C29R54 | 1115041 | 1115109 | 69 |
| 29 | C29R55 | 1115149 | 1115213 | 65 |
| 29 | C29R56 | 1115171 | 1115278 | 108 |
| 29 | C29R57 | 1190015 | 1189937 | 79 |
| 29 | C29R58 | 1205971 | 1205844 | 128 |
| 29 | C29R59 | 1206079 | 1205920 | 160 |
| 29 | C29R6 | 90536 | 90619 | 84 |
| 29 | C29R60 | 1206085 | 1206010 | 76 |
| 29 | C29R61 | 1206268 | 1206087 | 182 |
| 29 | C29R62 | 1217354 | 1217410 | 57 |
| 29 | C29R63 | 1217421 | 1217487 | 67 |
| 29 | C29R64 | 1217485 | 1217553 | 69 |
| 29 | C29R7 | 130990 | 131106 | 117 |
| 29 | C29R8 | 131121 | 131233 | 113 |
| 30 | C30R1 | 54458 | 54507 | 50 |
| 30 | C30R2 | 396473 | 396522 | 50 |
| 31 | C31R1 | 54478 | 54544 | 67 |
| 31 | C31R2 | 478676 | 478627 | 50 |
| 31 | C31R3 | 980839 | 980789 | 51 |
| 31 | C31R4 | 1230723 | 1230789 | 67 |
| 32 | C32R1 | 54656 | 54578 | 79 |
| 32 | C32R2 | 170918 | 170972 | 55 |
| 32 | C32R3 | 170922 | 171000 | 79 |
| 32 | C32R4 | 943559 | 943505 | 55 |
| 33 | C33R1 | 54817 | 54761 | 57 |
| 33 | C33R2 | 130564 | 130620 | 57 |
| 34 | C34R1 | 54842 | 54901 | 60 |
| 34 | C34R2 | 67944 | 68003 | 60 |
| 35 | C35R1 | 61246 | 62430 | 1185 |
| 35 | C35R10 | 165816 | 165728 | 89 |
| 35 | C35R11 | 166845 | 165819 | 1027 |
| 35 | C35R12 | 189963 | 191111 | 1149 |
| 35 | C35R13 | 236297 | 235150 | 1148 |
| 35 | C35R14 | 278049 | 277874 | 176 |
| 35 | C35R15 | 278243 | 278051 | 193 |
| 35 | C35R16 | 278351 | 278203 | 149 |
| 35 | C35R17 | 278449 | 278319 | 131 |
| 35 | C35R18 | 278485 | 278384 | 102 |
| 35 | C35R19 | 278612 | 278504 | 109 |
| 35 | C35R2 | 74289 | 75479 | 1191 |
| 35 | C35R20 | 278795 | 278583 | 213 |
| 35 | C35R21 | 279076 | 278797 | 280 |
| 35 | C35R22 | 326502 | 325565 | 938 |
| 35 | C35R23 | 326824 | 326502 | 323 |
| 35 | C35R24 | 352441 | 351506 | 936 |
| 35 | C35R25 | 352715 | 351744 | 972 |
| 35 | C35R26 | 510926 | 510838 | 89 |
| 35 | C35R27 | 511158 | 510928 | 231 |
| 35 | C35R28 | 511588 | 511160 | 429 |
| 35 | C35R29 | 511804 | 511399 | 406 |
| 35 | C35R3 | 75412 | 75490 | 79 |
| 35 | C35R30 | 512040 | 511761 | 280 |
| 35 | C35R31 | 512051 | 511928 | 124 |
| 35 | C35R32 | 539717 | 539639 | 79 |
| 35 | C35R33 | 539891 | 539719 | 173 |
| 35 | C35R34 | 540009 | 539776 | 234 |
| 35 | C35R35 | 540390 | 539969 | 422 |
| 35 | C35R36 | 540478 | 540201 | 278 |
| 35 | C35R37 | 540852 | 540392 | 461 |
| 35 | C35R38 | 635582 | 635866 | 285 |
| 35 | C35R39 | 635868 | 636145 | 278 |
| 35 | C35R4 | 80237 | 80994 | 758 |
| 35 | C35R40 | 635956 | 636188 | 233 |
| 35 | C35R41 | 636123 | 636253 | 131 |
| 35 | C35R42 | 636147 | 636384 | 238 |
| 35 | C35R43 | 636337 | 636529 | 193 |
| 35 | C35R44 | 636386 | 636616 | 231 |
| 35 | C35R45 | 636454 | 636626 | 173 |
| 35 | C35R46 | 636531 | 636705 | 175 |
| 35 | C35R47 | 845840 | 846301 | 462 |
| 35 | C35R48 | 845885 | 846371 | 487 |
| 35 | C35R49 | 846087 | 846492 | 406 |
| 35 | C35R5 | 80996 | 81180 | 185 |
| 35 | C35R50 | 846303 | 846535 | 233 |
| 35 | C35R51 | 846373 | 847054 | 682 |
| 35 | C35R52 | 963202 | 964158 | 957 |
| 35 | C35R53 | 964160 | 964398 | 239 |
| 35 | C35R6 | 81205 | 81350 | 146 |
| 35 | C35R7 | 107197 | 106518 | 680 |
| 35 | C35R8 | 107685 | 107199 | 487 |
| 35 | C35R9 | 165739 | 165648 | 92 |
| 36 | C36R1 | 67630 | 67681 | 52 |
| 36 | C36R2 | 178674 | 178623 | 52 |
| 37 | C37R1 | 68030 | 68083 | 54 |
| 37 | C37R2 | 68153 | 68206 | 54 |
| 38 | C38R1 | 72154 | 72204 | 51 |
| 38 | C38R10 | 980681 | 980799 | 119 |
| 38 | C38R11 | 980727 | 980821 | 95 |
| 38 | C38R12 | 1068289 | 1068238 | 52 |
| 38 | C38R13 | 1081121 | 1080969 | 153 |
| 38 | C38R14 | 1081170 | 1081121 | 50 |
| 38 | C38R15 | 1081184 | 1081133 | 52 |
| 38 | C38R16 | 1081216 | 1081157 | 60 |
| 38 | C38R17 | 1081235 | 1081171 | 65 |
| 38 | C38R18 | 1081296 | 1081237 | 60 |
| 38 | C38R19 | 1081308 | 1081251 | 58 |
| 38 | C38R2 | 462592 | 462489 | 104 |
| 38 | C38R20 | 1081355 | 1081275 | 81 |
| 38 | C38R21 | 1081402 | 1081306 | 97 |
| 38 | C38R22 | 1081414 | 1081357 | 58 |
| 38 | C38R23 | 1081473 | 1081370 | 104 |
| 38 | C38R24 | 1081482 | 1081381 | 102 |
| 38 | C38R25 | 1081507 | 1081411 | 97 |
| 38 | C38R26 | 1081526 | 1081462 | 65 |
| 38 | C38R27 | 1081536 | 1081486 | 51 |
| 38 | C38R28 | 1081638 | 1081556 | 83 |
| 38 | C38R29 | 1082970 | 1082918 | 53 |
| 38 | C38R3 | 691356 | 693415 | 2060 |
| 38 | C38R30 | 1247929 | 1247819 | 111 |
| 38 | C38R4 | 693325 | 693443 | 119 |
| 38 | C38R5 | 714403 | 714334 | 70 |
| 38 | C38R6 | 714456 | 714405 | 52 |
| 38 | C38R7 | 979185 | 979237 | 53 |
| 38 | C38R8 | 980529 | 980611 | 83 |
| 38 | C38R9 | 980660 | 980730 | 71 |
| 39 | C39R1 | 89117 | 89251 | 135 |
| 39 | C39R10 | 89945 | 90045 | 101 |
| 39 | C39R11 | 89971 | 90120 | 150 |
| 39 | C39R2 | 89256 | 89396 | 141 |
| 39 | C39R3 | 89397 | 89536 | 140 |
| 39 | C39R4 | 89425 | 89574 | 150 |
| 39 | C39R5 | 89538 | 89677 | 140 |
| 39 | C39R6 | 89566 | 89715 | 150 |
| 39 | C39R7 | 89679 | 89819 | 141 |
| 39 | C39R8 | 89804 | 89904 | 101 |
| 39 | C39R9 | 89906 | 89969 | 64 |
| 40 | C40R1 | 1173828 | 1173887 | 60 |
| 40 | C40R2 | 1175315 | 1175374 | 60 |
| 41 | C41R1 | 1094391 | 1094446 | 56 |
| 41 | C41R2 | 1217680 | 1217735 | 56 |
| 42 | C42R1 | 92740 | 92813 | 74 |
| 42 | C42R10 | 1103029 | 1101692 | 1338 |
| 42 | C42R2 | 92903 | 93081 | 179 |
| 42 | C42R3 | 93083 | 93241 | 159 |
| 42 | C42R4 | 93115 | 93735 | 621 |
| 42 | C42R5 | 93737 | 94011 | 275 |
| 42 | C42R6 | 512210 | 512052 | 159 |
| 42 | C42R7 | 512534 | 512180 | 355 |
| 42 | C42R8 | 512553 | 512480 | 74 |
| 42 | C42R9 | 1052477 | 1053814 | 1338 |
| 43 | C43R1 | 102854 | 102964 | 111 |
| 43 | C43R2 | 440346 | 440456 | 111 |
| 43 | C43R3 | 882231 | 882163 | 69 |
| 43 | C43R4 | 882267 | 882175 | 93 |
| 43 | C43R5 | 946312 | 946244 | 69 |
| 44 | C44R1 | 102965 | 103016 | 52 |
| 44 | C44R10 | 741940 | 741880 | 61 |
| 44 | C44R11 | 742212 | 741942 | 271 |
| 44 | C44R12 | 861502 | 861840 | 339 |
| 44 | C44R13 | 913491 | 913163 | 329 |
| 44 | C44R14 | 946457 | 946350 | 108 |
| 44 | C44R15 | 946631 | 946363 | 269 |
| 44 | C44R16 | 946692 | 946422 | 271 |
| 44 | C44R17 | 946709 | 946633 | 77 |
| 44 | C44R18 | 998108 | 997981 | 128 |
| 44 | C44R19 | 998178 | 998042 | 137 |
| 44 | C44R2 | 103018 | 103085 | 68 |
| 44 | C44R20 | 998229 | 998180 | 50 |
| 44 | C44R21 | 1000018 | 999960 | 59 |
| 44 | C44R22 | 1000186 | 999972 | 215 |
| 44 | C44R23 | 1000261 | 1000048 | 214 |
| 44 | C44R24 | 1000321 | 1000245 | 77 |
| 44 | C44R25 | 1129936 | 1129610 | 327 |
| 44 | C44R3 | 103087 | 103245 | 159 |
| 44 | C44R4 | 196648 | 196283 | 366 |
| 44 | C44R5 | 247780 | 247966 | 187 |
| 44 | C44R6 | 247843 | 248111 | 269 |
| 44 | C44R7 | 396368 | 396155 | 214 |
| 44 | C44R8 | 396435 | 396295 | 141 |
| 44 | C44R9 | 432176 | 432544 | 369 |
| 45 | C45R1 | 1094222 | 1094276 | 55 |
| 45 | C45R2 | 1115067 | 1115121 | 55 |
| 46 | C46R1 | 103291 | 103340 | 50 |
| 46 | C46R2 | 440448 | 440497 | 50 |
| 47 | C47R1 | 103306 | 103361 | 56 |
| 47 | C47R2 | 946255 | 946200 | 56 |
| 48 | C48R1 | 106188 | 106117 | 72 |
| 48 | C48R10 | 896194 | 896137 | 58 |
| 48 | C48R11 | 943437 | 943515 | 79 |
| 48 | C48R12 | 943443 | 943530 | 88 |
| 48 | C48R13 | 979026 | 978974 | 53 |
| 48 | C48R14 | 1035865 | 1035952 | 88 |
| 48 | C48R2 | 168538 | 168587 | 50 |
| 48 | C48R3 | 229619 | 229668 | 50 |
| 48 | C48R4 | 290664 | 290742 | 79 |
| 48 | C48R5 | 290683 | 290747 | 65 |
| 48 | C48R6 | 290696 | 290753 | 58 |
| 48 | C48R7 | 394072 | 394143 | 72 |
| 48 | C48R8 | 418150 | 418214 | 65 |
| 48 | C48R9 | 418165 | 418217 | 53 |
| 49 | C49R1 | 106251 | 106319 | 69 |
| 49 | C49R2 | 915297 | 915365 | 69 |
| 50 | C50R1 | 109229 | 107774 | 1456 |
| 50 | C50R2 | 237724 | 239187 | 1464 |
| 50 | C50R3 | 549459 | 548017 | 1443 |
| 51 | C51R1 | 118854 | 118909 | 56 |
| 51 | C51R2 | 527919 | 527860 | 60 |
| 51 | C51R3 | 527922 | 527867 | 56 |
| 51 | C51R4 | 854834 | 854893 | 60 |
| 52 | C52R1 | 123177 | 123226 | 50 |
| 52 | C52R2 | 1216442 | 1216491 | 50 |
| 53 | C53R1 | 1094054 | 1094110 | 57 |
| 53 | C53R2 | 1217343 | 1217399 | 57 |
| 54 | C54R1 | 123602 | 123669 | 68 |
| 54 | C54R2 | 290588 | 290655 | 68 |
| 55 | C55R1 | 123678 | 123727 | 50 |
| 55 | C55R2 | 1080951 | 1081000 | 50 |
| 56 | C56R1 | 130796 | 130863 | 68 |
| 56 | C56R2 | 1166488 | 1166555 | 68 |
| 57 | C57R1 | 130986 | 131038 | 53 |
| 57 | C57R2 | 418133 | 418081 | 53 |
| 58 | C58R1 | 135469 | 135526 | 58 |
| 58 | C58R2 | 135829 | 135886 | 58 |
| 59 | C59R1 | 138304 | 138413 | 110 |
| 59 | C59R2 | 139051 | 139160 | 110 |
| 60 | C60R1 | 151322 | 151248 | 75 |
| 60 | C60R2 | 418088 | 418161 | 74 |
| 60 | C60R3 | 462421 | 462496 | 76 |
| 61 | C61R1 | 158319 | 158249 | 71 |
| 61 | C61R2 | 196624 | 196694 | 71 |
| 62 | C62R1 | 158373 | 158432 | 60 |
| 62 | C62R2 | 162943 | 162999 | 57 |
| 62 | C62R3 | 473703 | 473789 | 87 |
| 62 | C62R4 | 473734 | 473793 | 60 |
| 62 | C62R5 | 772159 | 772073 | 87 |
| 62 | C62R6 | 1022670 | 1022728 | 59 |
| 63 | C63R1 | 1035727 | 1035810 | 84 |
| 63 | C63R2 | 1068035 | 1068118 | 84 |
| 64 | C64R1 | 170917 | 170802 | 116 |
| 64 | C64R2 | 170936 | 170866 | 71 |
| 64 | C64R3 | 679494 | 679564 | 71 |
| 64 | C64R4 | 1190494 | 1190609 | 116 |
| 65 | C65R1 | 170969 | 171026 | 58 |
| 65 | C65R2 | 1093944 | 1094001 | 58 |
| 66 | C66R1 | 196227 | 196178 | 50 |
| 66 | C66R2 | 1036070 | 1036119 | 50 |
| 67 | C67R1 | 196284 | 196229 | 56 |
| 67 | C67R2 | 369101 | 369041 | 61 |
| 67 | C67R3 | 369104 | 369049 | 56 |
| 67 | C67R4 | 395895 | 395955 | 61 |
| 67 | C67R5 | 832914 | 832964 | 51 |
| 68 | C68R1 | 200655 | 200586 | 70 |
| 68 | C68R2 | 229540 | 229609 | 70 |
| 69 | C69R1 | 200622 | 200701 | 80 |
| 69 | C69R2 | 385610 | 385689 | 80 |
| 70 | C70R1 | 200757 | 200689 | 69 |
| 70 | C70R2 | 794120 | 794188 | 69 |
| 71 | C71R1 | 226804 | 227253 | 450 |
| 71 | C71R10 | 848166 | 847858 | 309 |
| 71 | C71R11 | 865362 | 869310 | 3949 |
| 71 | C71R12 | 874607 | 875211 | 605 |
| 71 | C71R13 | 896259 | 896567 | 309 |
| 71 | C71R14 | 897484 | 897786 | 303 |
| 71 | C71R15 | 897575 | 898179 | 605 |
| 71 | C71R16 | 897827 | 898218 | 392 |
| 71 | C71R17 | 904671 | 908619 | 3949 |
| 71 | C71R18 | 1068653 | 1068475 | 179 |
| 71 | C71R19 | 1068984 | 1068655 | 330 |
| 71 | C71R2 | 226882 | 227482 | 601 |
| 71 | C71R20 | 1069098 | 1068967 | 132 |
| 71 | C71R21 | 1069549 | 1069100 | 450 |
| 71 | C71R3 | 227479 | 227870 | 392 |
| 71 | C71R4 | 309900 | 309782 | 119 |
| 71 | C71R5 | 310070 | 309902 | 169 |
| 71 | C71R6 | 310576 | 309976 | 601 |
| 71 | C71R7 | 723506 | 723455 | 52 |
| 71 | C71R8 | 723690 | 723633 | 58 |
| 71 | C71R9 | 848144 | 847771 | 374 |
| 72 | C72R1 | 227857 | 227920 | 64 |
| 72 | C72R2 | 908579 | 908642 | 64 |
| 73 | C73R1 | 229557 | 229508 | 50 |
| 73 | C73R2 | 432530 | 432595 | 66 |
| 73 | C73R3 | 919794 | 919729 | 66 |
| 73 | C73R4 | 1166515 | 1166575 | 61 |
| 73 | C73R5 | 1166522 | 1166589 | 68 |
| 73 | C73R6 | 1248183 | 1248123 | 61 |
| 74 | C74R1 | 229635 | 229585 | 51 |
| 74 | C74R2 | 349658 | 349708 | 51 |
| 75 | C75R1 | 229590 | 229644 | 55 |
| 75 | C75R2 | 396483 | 396537 | 55 |
| 76 | C76R1 | 1030543 | 1030598 | 56 |
| 76 | C76R2 | 1032331 | 1032386 | 56 |
| 77 | C77R1 | 241213 | 241263 | 51 |
| 77 | C77R2 | 608400 | 608450 | 51 |
| 78 | C78R1 | 241415 | 241464 | 50 |
| 78 | C78R2 | 608596 | 608645 | 50 |
| 79 | C79R1 | 241517 | 241568 | 52 |
| 79 | C79R2 | 611747 | 611798 | 52 |
| 80 | C80R1 | 242639 | 242759 | 121 |
| 80 | C80R2 | 609789 | 609909 | 121 |
| 81 | C81R1 | 245752 | 243822 | 1931 |
| 81 | C81R2 | 488449 | 486532 | 1918 |
| 81 | C81R3 | 507928 | 507468 | 461 |
| 81 | C81R4 | 510311 | 508845 | 1467 |
| 81 | C81R5 | 582552 | 584483 | 1932 |
| 81 | C81R6 | 634665 | 633946 | 720 |
| 82 | C82R1 | 255773 | 255843 | 71 |
| 82 | C82R2 | 580042 | 580112 | 71 |
| 83 | C83R1 | 264805 | 264750 | 56 |
| 83 | C83R2 | 630364 | 630419 | 56 |
| 84 | C84R1 | 267769 | 267714 | 56 |
| 84 | C84R2 | 627659 | 627714 | 56 |
| 85 | C85R1 | 267970 | 267894 | 77 |
| 85 | C85R2 | 627458 | 627534 | 77 |
| 86 | C86R1 | 279085 | 279154 | 70 |
| 86 | C86R2 | 528099 | 528030 | 70 |
| 87 | C87R1 | 279127 | 279177 | 51 |
| 87 | C87R2 | 1068117 | 1068167 | 51 |
| 88 | C88R1 | 279187 | 279236 | 50 |
| 88 | C88R2 | 1247722 | 1247771 | 50 |
| 89 | C89R1 | 286800 | 286888 | 89 |
| 89 | C89R2 | 901533 | 901624 | 92 |
| 89 | C89R3 | 916668 | 916609 | 60 |
| 90 | C90R1 | 290453 | 290534 | 82 |
| 90 | C90R2 | 1190202 | 1190269 | 68 |
| 90 | C90R3 | 1230498 | 1230578 | 81 |
| 91 | C91R1 | 290658 | 290711 | 54 |
| 91 | C91R2 | 792071 | 792124 | 54 |
| 92 | C92R1 | 290786 | 290843 | 58 |
| 92 | C92R2 | 752641 | 752584 | 58 |
| 93 | C93R1 | 293104 | 293154 | 51 |
| 93 | C93R2 | 1190451 | 1190501 | 51 |
| 94 | C94R1 | 293220 | 293170 | 51 |
| 94 | C94R2 | 792264 | 792314 | 51 |
| 95 | C95R1 | 308447 | 308518 | 72 |
| 95 | C95R2 | 456024 | 456095 | 72 |
| 96 | C96R1 | 308702 | 308640 | 63 |
| 96 | C96R2 | 901845 | 901907 | 63 |
| 97 | C97R1 | 314231 | 314293 | 63 |
| 97 | C97R2 | 314777 | 314839 | 63 |
| 98 | C98R1 | 339364 | 339429 | 66 |
| 98 | C98R2 | 997556 | 997607 | 52 |
| 98 | C98R3 | 1190221 | 1190286 | 66 |
| 99 | C99R1 | 339689 | 339741 | 53 |
| 99 | C99R2 | 1036353 | 1036405 | 53 |
| 100 | C100R1 | 368022 | 368079 | 58 |
| 100 | C100R2 | 368130 | 368187 | 58 |
| 101 | C101R1 | 369006 | 369083 | 78 |
| 101 | C101R2 | 1036111 | 1036034 | 78 |
| 102 | C102R1 | 376447 | 376496 | 50 |
| 102 | C102R2 | 1208681 | 1208730 | 50 |
| 103 | C103R1 | 379857 | 379934 | 78 |
| 103 | C103R2 | 379883 | 379943 | 61 |
| 103 | C103R3 | 839725 | 839785 | 61 |
| 103 | C103R4 | 1068373 | 1068296 | 78 |
| 104 | C104R1 | 379895 | 379948 | 54 |
| 104 | C104R2 | 1068415 | 1068362 | 54 |
| 105 | C105R1 | 380053 | 379974 | 80 |
| 105 | C105R2 | 854858 | 854937 | 80 |
| 106 | C106R1 | 385845 | 385950 | 106 |
| 106 | C106R2 | 528054 | 528159 | 106 |
| 107 | C107R1 | 385952 | 386095 | 144 |
| 107 | C107R2 | 528161 | 528304 | 144 |
| 108 | C108R1 | 386107 | 386204 | 98 |
| 108 | C108R2 | 528316 | 528413 | 98 |
| 109 | C109R1 | 387952 | 388004 | 53 |
| 109 | C109R2 | 912935 | 912987 | 53 |
| 110 | C110R1 | 388091 | 388153 | 63 |
| 110 | C110R2 | 1035901 | 1035839 | 63 |
| 111 | C111R1 | 388123 | 388173 | 51 |
| 111 | C111R2 | 388278 | 388328 | 51 |
| 112 | C112R1 | 388284 | 388343 | 60 |
| 112 | C112R2 | 1115002 | 1115061 | 60 |
| 113 | C113R1 | 418172 | 418227 | 56 |
| 113 | C113R2 | 730953 | 730898 | 56 |
| 114 | C114R1 | 438900 | 438958 | 59 |
| 114 | C114R2 | 1152534 | 1152592 | 59 |
| 115 | C115R1 | 458134 | 458079 | 56 |
| 115 | C115R2 | 1230710 | 1230765 | 56 |
| 116 | C116R1 | 458090 | 458152 | 63 |
| 116 | C116R2 | 752616 | 752678 | 63 |
| 117 | C117R1 | 458104 | 458173 | 70 |
| 117 | C117R2 | 792107 | 792038 | 70 |
| 118 | C118R1 | 462764 | 462710 | 55 |
| 118 | C118R2 | 1050821 | 1050875 | 55 |
| 119 | C119R1 | 473747 | 473797 | 51 |
| 119 | C119R2 | 997823 | 997773 | 51 |
| 120 | C120R1 | 478934 | 479001 | 68 |
| 120 | C120R2 | 478945 | 479049 | 105 |
| 120 | C120R3 | 478996 | 479060 | 65 |
| 120 | C120R4 | 864526 | 864422 | 105 |
| 120 | C120R5 | 1217586 | 1217653 | 68 |
| 120 | C120R6 | 1217649 | 1217713 | 65 |
| 121 | C121R1 | 484609 | 484660 | 52 |
| 121 | C121R2 | 912951 | 913002 | 52 |
| 122 | C122R1 | 506202 | 506272 | 71 |
| 122 | C122R2 | 506301 | 506371 | 71 |
| 123 | C123R1 | 512602 | 512552 | 51 |
| 123 | C123R2 | 1052470 | 1052520 | 51 |
| 124 | C124R1 | 525241 | 525302 | 62 |
| 124 | C124R2 | 953722 | 953783 | 62 |
| 125 | C125R1 | 525382 | 525495 | 114 |
| 125 | C125R2 | 953827 | 953940 | 114 |
| 126 | C126R1 | 538235 | 538179 | 57 |
| 126 | C126R2 | 541046 | 541153 | 108 |
| 126 | C126R3 | 687022 | 687131 | 110 |
| 126 | C126R4 | 744411 | 744358 | 54 |
| 126 | C126R5 | 1069674 | 1069740 | 67 |
| 127 | C127R1 | 547308 | 547249 | 60 |
| 127 | C127R2 | 775427 | 775481 | 55 |
| 127 | C127R3 | 1238660 | 1238721 | 62 |
| 128 | C128R1 | 676543 | 676872 | 330 |
| 128 | C128R2 | 809140 | 809469 | 330 |
| 129 | C129R1 | 676960 | 677067 | 108 |
| 129 | C129R2 | 809557 | 809664 | 108 |
| 130 | C130R1 | 678253 | 678308 | 56 |
| 130 | C130R2 | 913107 | 913162 | 56 |
| 131 | C131R1 | 678366 | 678314 | 53 |
| 131 | C131R2 | 1093837 | 1093889 | 53 |
| 132 | C132R1 | 679414 | 679470 | 57 |
| 132 | C132R2 | 946179 | 946235 | 57 |
| 133 | C133R1 | 679428 | 679490 | 63 |
| 133 | C133R2 | 679438 | 679493 | 56 |
| 133 | C133R3 | 772247 | 772192 | 56 |
| 133 | C133R4 | 882114 | 882174 | 61 |
| 133 | C133R5 | 882119 | 882210 | 92 |
| 133 | C133R6 | 919861 | 919805 | 57 |
| 133 | C133R7 | 1056725 | 1056816 | 92 |
| 134 | C134R1 | 679623 | 679673 | 51 |
| 134 | C134R2 | 792383 | 792433 | 51 |
| 135 | C135R1 | 693496 | 693445 | 52 |
| 135 | C135R2 | 714299 | 714366 | 68 |
| 135 | C135R3 | 714314 | 714369 | 56 |
| 135 | C135R4 | 980919 | 980864 | 56 |
| 135 | C135R5 | 1247729 | 1247796 | 68 |
| 136 | C136R1 | 694026 | 693972 | 55 |
| 136 | C136R2 | 1216447 | 1216501 | 55 |
| 137 | C137R1 | 714473 | 714408 | 66 |
| 137 | C137R2 | 980833 | 980898 | 66 |
| 138 | C138R1 | 731051 | 731114 | 64 |
| 138 | C138R2 | 1094065 | 1094128 | 64 |
| 139 | C139R1 | 742288 | 742230 | 59 |
| 139 | C139R2 | 913077 | 913135 | 59 |
| 140 | C140R1 | 758245 | 758324 | 80 |
| 140 | C140R2 | 758475 | 758554 | 80 |
| 141 | C141R1 | 758326 | 758390 | 65 |
| 141 | C141R2 | 758556 | 758620 | 65 |
| 142 | C142R1 | 777024 | 777101 | 78 |
| 142 | C142R2 | 964539 | 964462 | 78 |
| 143 | C143R1 | 777168 | 777103 | 66 |
| 143 | C143R2 | 964395 | 964460 | 66 |
| 144 | C144R1 | 792395 | 792444 | 50 |
| 144 | C144R2 | 1233965 | 1234014 | 50 |
| 145 | C145R1 | 1022748 | 1022797 | 50 |
| 145 | C145R2 | 1069582 | 1069631 | 50 |
| 146 | C146R1 | 811780 | 811727 | 54 |
| 146 | C146R2 | 1036433 | 1036486 | 54 |
| 147 | C147R1 | 832946 | 833020 | 75 |
| 147 | C147R2 | 1217152 | 1217226 | 75 |
| 148 | C148R1 | 833040 | 833090 | 51 |
| 148 | C148R2 | 1036154 | 1036204 | 51 |
| 149 | C149R1 | 839810 | 839867 | 58 |
| 149 | C149R2 | 1257037 | 1257094 | 58 |
| 150 | C150R1 | 845735 | 845796 | 62 |
| 150 | C150R2 | 978980 | 979041 | 62 |
| 151 | C151R1 | 847051 | 847760 | 710 |
| 151 | C151R2 | 872980 | 873689 | 710 |
| 151 | C151R3 | 1027711 | 1027770 | 60 |
| 152 | C152R1 | 848545 | 848594 | 50 |
| 152 | C152R2 | 1035957 | 1035908 | 50 |
| 153 | C153R1 | 882234 | 882285 | 52 |
| 153 | C153R2 | 1056840 | 1056891 | 52 |
| 154 | C154R1 | 896177 | 896101 | 77 |
| 154 | C154R2 | 1217015 | 1217091 | 77 |
| 155 | C155R1 | 913452 | 913508 | 57 |
| 155 | C155R2 | 998185 | 998241 | 57 |
| 156 | C156R1 | 943417 | 943467 | 51 |
| 156 | C156R2 | 1230690 | 1230740 | 51 |
| 157 | C157R1 | 998872 | 998961 | 90 |
| 157 | C157R2 | 999136 | 999225 | 90 |
| 158 | C158R1 | 999071 | 999149 | 79 |
| 158 | C158R2 | 999209 | 999287 | 79 |
