# Supplementary material for: Phylogenomics of the Reproductive Parasite Wolbachia pipientis wMel: A Streamlined Genome Overrun by Mobile Genetic Elements
Source: PLoS Biol. 2004 Mar 16;2(3):e69. doi: 10.1371/journal.pbio.0020069 (PMC368164; doi:10.1371/journal.pbio.0020069)
Supplement: Table S2 — (147 KB DOC). [file pbio.0020069.st002.doc]

Table S2. Inactivated genes in the *w*Mel genome.

| WD # | Type of inactivation[[1]](#endnote-2) | Start | Stop | Annotation | Role category |
| --- | --- | --- | --- | --- | --- |
| WD0257 | Truncation | 243803 | 243507 | DNA repair protein RadC, truncation | DNA metabolism |
| WD0507 | Truncation | 488804 | 488400 | DNA repair protein RadC, truncation | DNA metabolism |
| WD0277 | Truncation | 261085 | 261225 | conserved hypothetical protein, truncation | Hypothetical proteins |
| WD0562 | Truncation | 547952 | 547734 | transposase, truncation | Mobile and extrachromosomal element functions |
| WD0645 | Truncation | 634537 | 634061 | reverse transcriptase, truncation | Mobile and extrachromosomal element functions |
| WD0841 | Truncation | 808154 | 807855 | transposase, IS5 family, truncation | Mobile and extrachromosomal element functions |
| WD0874 | Truncation | 847696 | 847001 | transposase, truncation | Mobile and extrachromosomal element functions |
| WD0875 | Truncation | 848079 | 847786 | transposase, IS5 family, truncation | Mobile and extrachromosomal element functions |
| WD0947 | Truncation | 908139 | 908543 | transposase, IS5 family, truncation | Mobile and extrachromosomal element functions |
| WD0454 | Point mutations and frame shifts | 435704 | 437082 | amino acid ABC transporter, permease/substrate-binding protein, putative, authentic frameshift | Transport and binding proteins |
| WD0624 | Point mutations and frame shifts | 610444 | 609315 | conserved domain protein, authentic frameshift | Hypothetical proteins |
| WD0339 | Point mutations and frame shifts | 323334 | 322921 | conserved hypothetical protein, authentic point mutation | Hypothetical proteins |
| WD0718 | Point mutations and frame shifts | 692779 | 691331 | conserved hypothetical protein, authentic point mutation | Hypothetical proteins |
| WD0881 | Point mutations and frame shifts | 851349 | 851731 | dihydroneopterin aldolase, authentic frameshift | Biosynthesis of cofactors, prosthetic groups, and carriers |
| WD1032 | Point mutations and frame shifts | 992093 | 993276 | membrane protein, putative, authentic frameshift | Cell envelope |
| WD1300 | Point mutations and frame shifts | 1243240 | 1242304 | Na+/H+ antiporter subunit, putative, authentic frameshift | Transport and binding proteins |
| WD0374 | Point mutations and frame shifts | 353907 | 352806 | peptide chain release factor 2, authentic frameshift | Protein synthesis |
| WD0376 | Point mutations and frame shifts | 357146 | 355690 | potassium uptake protein TrkH, putative, authentic frameshift | Transport and binding proteins |
| WD0280 | Point mutations and frame shifts | 262193 | 262653 | prophage LambdaW1, baseplate assembly protein V, putative, authentic frameshift | Mobile and extrachromosomal element functions |
| WD0271 | Point mutations and frame shifts | 255335 | 256426 | prophage LambdaW1, minor capsid protein, authentic point mutation | Mobile and extrachromosomal element functions |
| WD0270 | Point mutations and frame shifts | 253852 | 255266 | prophage LambdaW1, portal protein, authentic frameshift | Mobile and extrachromosomal element functions |
| WD0272 | Point mutations and frame shifts | 256859 | 257868 | prophage LambdaW1, transposase, IS110 family, authentic frameshift | Mobile and extrachromosomal element functions |
| WD0601 | Point mutations and frame shifts | 578196 | 579610 | prophage LambdaW4, portal protein, lambda family, authentic frameshift | Mobile and extrachromosomal element functions |
| WD0575 | Point mutations and frame shifts | 558081 | 556898 | prophage P2W3, major tail sheath protein, putative, authentic frameshift | Mobile and extrachromosomal element functions |
| WD0422 | Point mutations and frame shifts | 399639 | 398177 | proton-dependent oligopeptide transport family protein, authentic frameshift | Transport and binding proteins |
| WD0258 | Point mutations and frame shifts | 245244 | 243935 | reverse transcriptase, authentic frameshift | Mobile and extrachromosomal element functions |
| WD0506 | Point mutations and frame shifts | 487954 | 486645 | reverse transcriptase, authentic frameshift | Mobile and extrachromosomal element functions |
| WD0606 | Point mutations and frame shifts | 583060 | 584369 | reverse transcriptase, authentic frameshift | Mobile and extrachromosomal element functions |
| WD0396 | Point mutations and frame shifts | 378869 | 377325 | reverse transcriptase, authentic point mutation | Mobile and extrachromosomal element functions |
| WD0538 | Point mutations and frame shifts | 523124 | 524668 | reverse transcriptase, authentic point mutation | Mobile and extrachromosomal element functions |
| WD0545 | Point mutations and frame shifts | 532234 | 529830 | TPR domain protein, authentic frameshift | Unknown function |
| WD0956 | Point mutations and frame shifts | 915501 | 916458 | transposase, IS110 family, authentic frameshift | Mobile and extrachromosomal element functions |
| WD0961 | Point mutations and frame shifts | 921236 | 921598 | transposase, IS5 family, OrfA, authentic point mutation | Mobile and extrachromosomal element functions |
| WD1224 | Interruptions | 1171707 | 1172285 | mannose-1-phosphate guanylyltransferase, interruption-N | Cell envelope |
| WD1227 | Interruptions | 1173145 | 1173915 | mannose-1-phosphate guanylyltransferase, interruption-C | Cell envelope |
| WD0918 | Interruptions | 885936 | 886244 | competence protein F, interruption-N | Cellular processes |
| WD0921 | Interruptions | 887143 | 887578 | competence protein F, interruption-C | Cellular processes |
| WD0261 | Interruptions | 247670 | 247807 | conserved hypothetical protein, interruption-N | Hypothetical proteins |
| WD0262 | Interruptions | 248155 | 248478 | conserved hypothetical protein, interruption-C | Hypothetical proteins |
| WD0043 | Interruptions | 46860 | 46177 | reverse transcriptase, interruption-C | Mobile and extrachromosomal element functions |
| WD0046 | Interruptions | 48759 | 47720 | reverse transcriptase, interruption-N | Mobile and extrachromosomal element functions |
| WD0515 | Interruptions | 507960 | 507583 | reverse transcriptase, interruption-C | Mobile and extrachromosomal element functions |
| WD0518 | Interruptions | 509811 | 508826 | reverse transcriptase, interruption-N | Mobile and extrachromosomal element functions |
| WD0900 | Interruptions | 865752 | 865478 | transposase, Tn5-related, interruption-C | Mobile and extrachromosomal element functions |
| WD0902 | Interruptions | 868076 | 867067 | transposase, Tn5-related, interruption-N | Mobile and extrachromosomal element functions |
| WD0932 | Interruptions | 896358 | 896603 | transposase, IS5 family, interruption-N | Mobile and extrachromosomal element functions |
| WD0935 | Interruptions | 897561 | 898170 | transposase, IS5 family, interruption-C | Mobile and extrachromosomal element functions |
| WD0943 | Interruptions | 905061 | 904657 | transposase, Tn5-related, interruption-C | Mobile and extrachromosomal element functions |
| WD0945 | Interruptions | 907385 | 906376 | transposase, Tn5-related, interruption-N | Mobile and extrachromosomal element functions |
| WD0126 | Degenerate | 116159 | 115890 | conserved hypothetical protein, degenerate | Hypothetical proteins |
| WD0275 | Degenerate | 259436 | 259693 | conserved hypothetical protein, degenerate | Hypothetical proteins |
| WD0593 | Degenerate | 571699 | 572142 | conserved hypothetical protein, degenerate | Hypothetical proteins |
| WD0834 | Degenerate | 799896 | 800664 | conserved hypothetical protein, degenerate | Hypothetical proteins |
| WD1131 | Degenerate | 1081547 | 1082119 | conserved hypothetical protein, degenerate | Hypothetical proteins |
| WD1164 | Degenerate | 1113542 | 1112766 | conserved hypothetical protein, degenerate | Hypothetical proteins |
| WD1214 | Degenerate | 1161812 | 1161045 | coproporphyrinogen III oxidase, aerobic, degenerate | Biosynthesis of cofactors, prosthetic groups, and carriers |
| WD0218 | Degenerate | 199489 | 199709 | portal protein, degenerate | Mobile and extrachromosomal element functions |
| WD0962 | Degenerate | 921813 | 922150 | reverse transcriptase, degenerate | Mobile and extrachromosomal element functions |
| WD0510 | Degenerate | 492288 | 491771 | ribonuclease, degenerate | Transcription |
| WD0050 | Degenerate | 51515 | 51261 | transposase, degenerate | Mobile and extrachromosomal element functions |
| WD0711 | Degenerate | 685645 | 686900 | transposase, degenerate | Mobile and extrachromosomal element functions |
| WD0842 | Degenerate | 808258 | 808882 | transposase, degenerate | Mobile and extrachromosomal element functions |
| WD0907 | Degenerate | 872362 | 872745 | transposase, degenerate | Mobile and extrachromosomal element functions |
| WD0908 | Degenerate | 873625 | 872777 | transposase, degenerate | Mobile and extrachromosomal element functions |
| WD0941 | Degenerate | 902705 | 901858 | transposase, degenerate | Mobile and extrachromosomal element functions |
| WD0698 | Degenerate | 675976 | 676767 | transposase, IS110 family, degenerate | Mobile and extrachromosomal element functions |
| WD0843 | Degenerate | 809082 | 809364 | transposase, IS110 family, degenerate | Mobile and extrachromosomal element functions |
| WD0901 | Degenerate | 865969 | 866897 | transposase, IS110 family, degenerate | Mobile and extrachromosomal element functions |
| WD0944 | Degenerate | 905278 | 906206 | transposase, IS110 family, degenerate | Mobile and extrachromosomal element functions |
| WD1296 | Degenerate | 1238039 | 1238630 | transposase, IS110 family, degenerate | Mobile and extrachromosomal element functions |
| WD0063 | Degenerate | 62366 | 61335 | transposase, IS3 family, degenerate | Mobile and extrachromosomal element functions |
| WD0081 | Degenerate | 75415 | 74384 | transposase, IS3 family, degenerate | Mobile and extrachromosomal element functions |
| WD0088 | Degenerate | 81314 | 80420 | transposase, IS3 family, degenerate | Mobile and extrachromosomal element functions |
| WD0114 | Degenerate | 106591 | 107639 | transposase, IS3 family, degenerate | Mobile and extrachromosomal element functions |
| WD0182 | Degenerate | 165723 | 166754 | transposase, IS3 family, degenerate | Mobile and extrachromosomal element functions |
| WD0207 | Degenerate | 191040 | 189997 | transposase, IS3 family, degenerate | Mobile and extrachromosomal element functions |
| WD0250 | Degenerate | 235221 | 236264 | transposase, IS3 family, degenerate | Mobile and extrachromosomal element functions |
| WD0298 | Degenerate | 277949 | 278842 | transposase, IS3 family, degenerate | Mobile and extrachromosomal element functions |
| WD0344 | Degenerate | 325641 | 326735 | transposase, IS3 family, degenerate | Mobile and extrachromosomal element functions |
| WD0372 | Degenerate | 351580 | 352620 | transposase, IS3 family, degenerate | Mobile and extrachromosomal element functions |
| WD0520 | Degenerate | 510913 | 511960 | transposase, IS3 family, degenerate | Mobile and extrachromosomal element functions |
| WD0554 | Degenerate | 539714 | 540762 | transposase, IS3 family, degenerate | Mobile and extrachromosomal element functions |
| WD0648 | Degenerate | 636631 | 635584 | transposase, IS3 family, degenerate | Mobile and extrachromosomal element functions |
| WD0873 | Degenerate | 846979 | 845931 | transposase, IS3 family, degenerate | Mobile and extrachromosomal element functions |
| WD1004 | Degenerate | 964322 | 963282 | transposase, IS3 family, degenerate | Mobile and extrachromosomal element functions |
| WD0240 | Degenerate | 226892 | 227814 | transposase, IS5 family, degenerate | Mobile and extrachromosomal element functions |
| WD0329 | Degenerate | 310566 | 309629 | transposase, IS5 family, degenerate | Mobile and extrachromosomal element functions |
| WD0336 | Degenerate | 319733 | 318984 | transposase, IS5 family, degenerate | Mobile and extrachromosomal element functions |
| WD0749 | Degenerate | 723618 | 722695 | transposase, IS5 family, degenerate | Mobile and extrachromosomal element functions |
| WD0903 | Degenerate | 868333 | 869234 | transposase, IS5 family, degenerate | Mobile and extrachromosomal element functions |
| WD0911 | Degenerate | 874611 | 875202 | transposase, IS5 family, degenerate | Mobile and extrachromosomal element functions |
| WD1114 | Degenerate | 1069461 | 1068524 | transposase, IS5 family, degenerate | Mobile and extrachromosomal element functions |
| WD0048 | Degenerate | 51019 | 49649 | transposase, Tn5-related, degenerate | Mobile and extrachromosomal element functions |
| WD0326 | Internal deletion | 308245 | 307334 | transposase, IS110 family, internal deletion | Mobile and extrachromosomal element functions |
|  |  |  |  |  |  |

1. Interruptions are cases in which you find both the amino- and carboxy-termini of a protein separated by some sequence such as a transposon. Truncations are ORFs in which some significant segment is missing. This should not include ORFs that are just a little shorter than database matches or ORFs that are simply a distinct domain. Authentic frameshifts and authentic point mutations are those ORFs that have a significant difference (that is confirmed by the sequencing laboratory) with database matches suggesting that they no longer encode fully functional proteins. Degenerate ORFs are those disrupted by multiple frameshifts and point mutations. Internal deletions are the absence of a region of DNA in the interior of an ORF relative to its orthologs. [↑](#endnote-ref-2)
