# Supplementary material for: Phylogenomics of the Reproductive Parasite Wolbachia pipientis wMel: A Streamlined Genome Overrun by Mobile Genetic Elements
Source: PLoS Biol. 2004 Mar 16;2(3):e69. doi: 10.1371/journal.pbio.0020069 (PMC368164; doi:10.1371/journal.pbio.0020069)
Supplement: Table S4 — (116 KB DOC). [file pbio.0020069.st004.doc]

Table S4. Putative lineage-specific gene duplications in *w*Mel. These were identified as those genes encoding proteins with better blastp matches to other proteins in *w*Mel compared to all other complete genomes.

| Family and Gene IDs | Annotation | | |
| --- | --- | --- | --- |
| Family 1. |  | | |
| WD0694 | conserved hypothetical protein | |  |
| WD0047 | conserved domain protein | |  |
| WD0395 | conserved hypothetical protein | |  |
| WD1137 | conserved hypothetical protein | |  |
| WD0296 | conserved hypothetical protein | |  |
| WD0539 | conserved hypothetical protein | |  |
| WD0042 | conserved hypothetical protein | |  |
| WD0996 | conserved hypothetical protein | |  |
| WD0511 | conserved hypothetical protein | |  |
| WD0180 | conserved domain protein | |  |
| WD0627 | conserved hypothetical protein | |  |
| WD0691 | conserved hypothetical protein | |  |
| WD0226 | conserved hypothetical protein | |  |
| WD0398 | conserved hypothetical protein | |  |
| WD1140 | conserved hypothetical protein | |  |
| WD0993 | conserved hypothetical protein | |  |
| WD0536 | conserved hypothetical protein | |  |
|  |  | | |
| Family 2. |  | | |
| WD0633 | prophage LambdaW5, ankyrin repeat domain protein | |  |
| WD0256 | hypothetical protein | |  |
| WD0292 | prophage LambdaW1, ankyrin repeat domain protein | |  |
| WD0443 | hypothetical protein | |  |
|  |  | | |
| Family 3. |  | | |
| WD0275 | conserved hypothetical protein, degenerate | |  |
| WD0605 | hypothetical protein | |  |
|  |  | | |
| Family 4. |  | | |
| WD0051 | hypothetical protein | |  |
| WD0898 | hypothetical protein | |  |
|  |  | | |
| Family 5. |  | | |
| WD0052 | hypothetical protein | |  |
| WD0310 | hypothetical protein | |  |
|  |  | |  |
| Family 6. |  | | |
| WD0634 | prophage LambdaW5, site-specific recombinase, resolvase family | |  |
| WD0288 | prophage LambdaW1, site-specific recombinase, resolvase family | |  |
|  |  | |  |
| Family 7. |  | | |
| WD0635 | conserved hypothetical protein | |  |
| WD0287 | conserved hypothetical protein | |  |
|  |  | |  |
| Family 8. |  | | |
| WD0278 | prophage LambdaW1, minor tail protein Z, putative | |  |
| WD0644 | prophage LambdaW5, minor tail protein Z, putative | |  |
|  |  | |  |
| Family 9. |  | | |
| WD0279 | conserved hypothetical protein | |  |
| WD0643 | conserved hypothetical protein | |  |
|  |  | |  |
| Family 10. |  | | |
| WD0582 | regulatory protein RepA, putative | |  |
| WD0609 | regulatory protein RepA, putative | |  |
|  |  | |  |
| Family 11. |  | | |
| WD0225 | hypothetical protein | |  |
| WD0397 | hypothetical protein | |  |
| WD1139 | hypothetical protein | |  |
| WD0994 | hypothetical protein | |  |
| WD0537 | hypothetical protein | |  |
| WD0692 | hypothetical protein | |  |
|  |  | |  |
| Family 12. |  | | |
| WD0932 | transposase, IS5 family, interruption-N | |  |
| WD0875 | transposase, IS5 family, truncation | |  |
|  |  | |  |
| Family 13. |  | | |
| WD0281 | hypothetical protein | |  |
| WD0641 | hypothetical protein | |  |
|  |  | |  |
| Family 14. |  | | |
| WD0282 | prophage LambdaW1, baseplate assembly protein W, putative | |  |
| WD0640 | prophage LambdaW5, baseplate assembly protein W, putative | |  |
|  |  | |  |
| Family 15. |  | | |
| WD0283 | prophage LambdaW1, baseplate assembly protein J, putative | |  |
| WD0639 | prophage LambdaW5, baseplate assembly protein J, putative | |  |
|  |  | |  |
| Family 16. |  | | |
| WD0231 | hypothetical protein | |  |
| WD0630 | hypothetical protein | |  |
|  |  | |  |
| Family 17. |  | | |
| WD0338 | hypothetical protein | |  |
| WD0823 | hypothetical protein | |  |
| WD0383 | hypothetical protein | |  |
|  |  | | |
| Family 18. |  | | |
| WD0522 | hypothetical protein | |  |
| WD1153 | hypothetical protein | |  |
| WD1095 | hypothetical protein | |  |
|  |  | | |
| Family 19. |  | | |
| WD0404 | conserved hypothetical protein | |  |
| WD0600 | conserved hypothetical protein | |  |
| WD0122 | conserved hypothetical protein | |  |
| WD0124 | conserved hypothetical protein | |  |
| WD0269 | conserved hypothetical protein | |  |
| WD0126 | conserved hypothetical protein, degenerate | |  |
|  |  | |  |
| Family 20. |  | | |
| WD1138 | reverse transcriptase, putative | |  |
| WD0995 | reverse transcriptase | |  |
| WD0693 | reverse transcriptase, putative | |  |
|  |  | |  |
| Family 21. |  | | |
| WD0696 | hypothetical protein | |  |
| WD0335 | hypothetical protein | |  |
|  |  | |  |
| Family 22. |  | | |
| WD0346 | Fic family protein | |  |
| WD0365 | Fic family protein | |  |
|  |  | |  |
| Family 23. |  | | |
| WD0110 | hypothetical protein | |  |
| WD0459 | hypothetical protein | |  |
|  |  | |  |
| Family 24. |  | | |
| WD0636 | prophage LambdaW5, ankyrin repeat domain protein | |  |
| WD0285 | prophage LambdaW1, ankyrin repeat domain protein | |  |
| WD0637 | prophage LambdaW5, ankyrin repeat domain protein | |  |
| WD0286 | prophage LambdaW1, ankyrin repeat domain protein | |  |
|  |  | | |
| Family 25. |  | | |
| WD0583 | hypothetical protein | |  |
| WD0586 | hypothetical protein | |  |
| WD0589 | hypothetical protein | |  |
|  |  | |  |
| Family 26. |  | | |
| WD0933 | transposase, IS5 family, OrfA | |  |
| WD0910 | transposase, IS5 family, OrfA | |  |
| WD0328 | transposase, IS5 family, OrfA | |  |
| WD0920 | transposase, IS5 family, OrfA | |  |
| WD0646 | transposase, IS5 family, OrfA | |  |
| WD0456 | transposase, IS5 family, OrfA | |  |
| WD0517 | transposase, IS5 family, OrfA | |  |
| WD0216 | transposase, IS5 family, OrfA | |  |
| WD0588 | transposase, IS5 family, OrfA | |  |
| WD0137 | transposase, IS5 family, OrfA | |  |
| WD0546 | transposase, IS5 family, OrfA | |  |
| WD1225 | transposase, IS5 family, OrfA | |  |
| WD0045 | transposase, IS5 family, OrfA | |  |
|  |  | |  |
| Family 27. |  | | |
| WD0638 | conserved hypothetical protein | |  |
| WD0284 | conserved hypothetical protein | |  |
|  |  | |  |
| Family 28. |  | | |
| WD0181 | HNH endonuclease family protein | |  |
| WD0043 | reverse transcriptase, interruption-C | |  |
|  |  | |  |
| Family 29. |  | | |
| WD0407 | Na+/H+ antiporter, putative | |  |
| WD0316 | Na+/H+ antiporter family protein | |  |
|  |  | |  |
| Family 30. |  | | |
| WD0410 | hypothetical protein | |  |
| WD1078 | hypothetical protein | |  |
|  |  | |  |
| Family 31. |  | | |
| WD0474 | hypothetical protein | |  |
| WD0651 | hypothetical protein | |  |
| WD1289 | hypothetical protein | |  |
| WD1290 | hypothetical protein | |  |
| WD0351 | hypothetical protein | |  |
| WD1288 | hypothetical protein | |  |
|  |  | |  |
| Family 32. |  | | |
| WD1152 | hypothetical protein |  | |
| WD0102 | hypothetical protein |  | |
| WD1096 | hypothetical protein |  | |
|  |  | |  |
| Family 33. |  | | |
| WD0115 | transposase, IS4 family | |  |
| WD0563 | transposase, IS4 family | |  |
| WD0252 | transposase, IS4 family | |  |
|  |  | |  |
| Family 34. |  | | |
| WD0645 | reverse transcriptase, truncation | |  |
| WD0515 | reverse transcriptase, interruption-C | |  |
|  |  | |  |
| Family 35. |  | | |
| WD0942 | hypothetical protein | |  |
| WD1228 | hypothetical protein | |  |
|  |  | |  |
| Family 36. |  | | |
| WD0295 | hypothetical protein | |  |
| WD0512 | hypothetical protein | |  |
|  |  | |  |
| Family 37. |  | | |
| WD1226 | transposase, IS5 family, OrfB | |  |
| WD0215 | transposase, IS5 family, OrfB | |  |
| WD0327 | transposase, IS5 family, OrfB | |  |
| WD0457 | transposase, IS5 family, OrfB | |  |
| WD0516 | transposase, IS5 family, OrfB | |  |
| WD0547 | transposase, IS5 family, OrfB | |  |
| WD0587 | transposase, IS5 family, OrfB | |  |
| WD0647 | transposase, IS5 family, OrfB | |  |
| WD0044 | transposase, IS5 family, OrfB | |  |
| WD0909 | transposase, IS5 family, OrfB | |  |
| WD0138 | transposase, IS5 family, OrfB | |  |
| WD0919 | transposase, IS5 family, OrfB | |  |
| WD0934 | transposase, IS5 family, OrfB | |  |
|  |  | |  |
| Family 38. |  | | |
| WD1033 | permease, putative | |  |
| WD1034 | membrane protein, putative | |  |
|  |  | |  |
| Family 39. |  | | |
| WD0594 | prophage LambdaW4, DNA methylase | |  |
| WD0263 | prophage LambdaW1, DNA methylase | |  |
|  |  | |  |
| Family 40. |  | | |
| WD0595 | conserved hypothetical protein | |  |
| WD0264 | conserved hypothetical protein | |  |
|  |  | |  |
| Family 41. |  | | |
| WD0597 | prophage LambdaW4, terminase large subunit, putative | |  |
| WD0265 | prophage LambdaW1, terminase large subunit, putative | |  |
|  |  | |  |
| Family 42. |  | | |
| WD0598 | hypothetical protein | |  |
| WD0266 | hypothetical protein | |  |
|  |  | |  |
| Family 43. |  | | |
| WD0125 | hypothetical protein | |  |
| WD0268 | hypothetical protein | |  |
|  |  | |  |
| Family 44. |  | | |
| WD0017 | translation elongation factor Tu (tuf-1) | |  |
| WD0683 | translation elongation factor Tu (tuf-2) | |  |
|  |  | |  |
| Family 45. |  | | |
| WD0899 | hypothetical protein | |  |
| WD0049 | hypothetical protein | |  |
|  |  | |  |
| Family 46. |  | | |
| WD0603 | conserved hypothetical protein | |  |
| WD0273 | conserved hypothetical protein | |  |
|  |  | |  |
| Family 47. |  | | |
| WD0418 | hypothetical protein | |  |
| WD0861 | hypothetical protein | |  |
| WD1276 | hypothetical protein | |  |
| WD0386 | hypothetical protein | |  |
|  |  | |  |
| Family 48. |  | | |
| WD0604 | conserved hypothetical protein | |  |
| WD0274 | conserved hypothetical protein | |  |
|  |  | |  |
| Family 49. |  | | |
| WD0784 | hypothetical protein | |  |
| WD0785 | hypothetical protein | |  |
|  |  | |  |
| Family 50. |  | | |
| WD0139 | TenA/THI-4 family protein | |  |
| WD0140 | transcriptional regulator, putative | |  |
|  |  | |  |
| Family 51. |  | | |
| WD0254 | transcriptional regulator |  | |
| WD0622 | transcriptional regulator |  | |
| WD0623 | transcriptional regulator |  | |
| WD0508 | transcriptional regulator |  | |
| WD0626 | transcriptional regulator |  | |
| WD0255 | transcriptional regulator |  | |
| WD0564 | hypothetical protein |  | |
|  |  | |  |
| Family 52. |  | | |
| WD1111 | hypothetical protein | |  |
| WD0332 | hypothetical protein | |  |
|  |  | |  |
| Family 53. |  | | |
| WD0498 | ankyrin repeat domain protein | |  |
| WD0035 | ankyrin repeat domain protein | |  |
|  |  | |  |
| Family 54. |  | | |
| WD0792 | hypothetical protein | |  |
| WD0793 | hypothetical protein | |  |
|  |  | | |
| Family 55. |  | | |
| WD0907 | transposase, degenerate | |  |
| WD0050 | transposase, degenerate | |  |
|  |  | |  |
| Family 56. |  | | |
| WD1118 | conserved hypothetical protein | |  |
| WD0209 | hypothetical protein | |  |
|  |  | |  |
| Family 57. |  | | |
| WD0033 | hypothetical protein | |  |
| WD0034 | hypothetical protein | |  |
|  |  | |  |
| Family 58. |  | | |
| WD0509 | DNA mismatch repair protein MutL-2 (mutL-2) | |  |
| WD1306 | DNA mismatch repair protein MutL-1 (mutL-1) | |  |
|  |  | |  |
| Family 59. |  | | |
| WD0150 | exopolysaccharide synthesis protein ExoD-related protein | |  |
| WD0151 | exopolysaccharide synthesis protein ExoD-related protein | |  |
|  |  | |  |
| Family 60. |  | | |
| WD0220 | hypothetical protein | |  |
| WD0809 | hypothetical protein | |  |
|  |  | |  |
| Family 61. |  | | |
| WD1072 | DnaJ domain protein |  | |
| WD1074 | DnaJ domain protein |  | |
