# Supplementary material for: Phylogenomics of the Reproductive Parasite Wolbachia pipientis wMel: A Streamlined Genome Overrun by Mobile Genetic Elements
Source: PLoS Biol. 2004 Mar 16;2(3):e69. doi: 10.1371/journal.pbio.0020069 (PMC368164; doi:10.1371/journal.pbio.0020069)
Supplement: Table S5 — (24 KB DOC). [file pbio.0020069.st005.doc]

**TableS5.** Genetic distances as calculated for alignments of *wsp* and *wspB* gene sequences from the same *Wolbachia* strains.

|  |  | ***wsp*** |  |  |
| --- | --- | --- | --- | --- |
|  |  |  |  |  |
|  | *w*Mel | *w*Ri | *w*AlbB | *D. immitis* |
| *w*Mel |  | 0.23 | 0.38 | 0.53 |
| *w*Ri | 0.32 |  | 0.3 | 0.55 |
| *w*AlbB | 0.36 | 0.23 |  | 0.49 |
| *D. immitis* | 0.67 | 0.84 | 0.75 |  |
|  |  |  |  |  |
|  |  | ***wspB*** |  |  |
|  |  |  |  |  |
