# Supplementary material for: Phylogenomics of the Reproductive Parasite Wolbachia pipientis wMel: A Streamlined Genome Overrun by Mobile Genetic Elements
Source: PLoS Biol. 2004 Mar 16;2(3):e69. doi: 10.1371/journal.pbio.0020069 (PMC368164; doi:10.1371/journal.pbio.0020069)
Supplement: Table S6 — (26 KB DOC). [file pbio.0020069.st006.doc]

Table S6. Putative DNA repair genes in the *w*Mel genome.

| Repair Category | Gene Name | ID |
| --- | --- | --- |
|  |  |  |
| Mismatch excision repair | *mutS1* | WD0190 |
|  | *mutL1* | WD0509 |
|  | *mutL2* | WD1306 |
|  |  |  |
| Nucleotide excision repair | *uvrA* | WD0916 |
|  | *uvrB* | WD0839 |
|  | *uvrC* | WD0154 |
|  | *uvrD* | WD0963 |
|  |  |  |
| Base excision repair | *fpg* | WD1158 |
|  | *mpg* | WD1110 |
|  | *nth* | WD0789 |
|  | *xth* | WD1001 |
|  |  |  |
| Homologous recombination | *recA* | WD1050 |
|  | *ruvA* | WD1112 |
|  | *ruvB* | WD1113 |
|  | *ruvC* | WD0142 |
|  | *recG* | WD0824 |
|  | *recR* | WD1180 |
|  | *recF* | WD1286 |
|  | *recJ* | WD0312 |
|  |  |  |
| Other | *ligA* | WD0776 |
|  | *ssb* | WD0774 |
|  | *radA* | WD0916 |
