# Supplementary material for: Phylogenomics of the Reproductive Parasite Wolbachia pipientis wMel: A Streamlined Genome Overrun by Mobile Genetic Elements
Source: PLoS Biol. 2004 Mar 16;2(3):e69. doi: 10.1371/journal.pbio.0020069 (PMC368164; doi:10.1371/journal.pbio.0020069)
Supplement: Table S7 — (34 KB DOC). [file pbio.0020069.st007.doc]

# Table S7. Phylogenetic results for concatenated data of 32 mitochondrial proteins

|  |  |  |  |  |  |  |  |  |
| --- | --- | --- | --- | --- | --- | --- | --- | --- |
|  | BP support for subtopologies T_s_1, T_s_2, T_s_3, and T_s_4 | | | | | | | |
| Method | 6776 sites ^a^ | | | | 3100 sites^b^ | | | |
|  | T_s_1 | T_s_2 | T_s_3 | T_s_4 | T_s_1 | T_s_2 | T_s_3 | T_s_4 |
| NJLogDet | 100 | 100 | 90 | n.d.^d^ | 100 | 86 | 11 | 89 |
| NJDayhoff | 100 | 100 | 100 | 0 | 96 | 100 | 64 | 36 |
| NJJTT | 100 | 100 | 100 | 0 | 95 | 100 | 50 | 49 |
| Protpars | 89 | 98 | 89 | 0 | 100 | 80 | 13 | 69 |
| Puzzle^c^ JTT | 98 | 100 | 100 | 0 | 100 | 93 | 85 | n.d. |
| Puzzle mtREV | 98 | 100 | 100 | 0 | 100 | 93 | 92 | n.d. |

Notes:

T_s_1: a common branch for *Reclinomonas* and *Marchantia* mitochondria

T_s_2: a common branch for *Wolbachia* and *Rickettsia*

T_s_3: a common branch for mitochondria with (*Wolbachia* and *Rickettsia*)

T_s_4: a common branch for mitochondria with free-living α-proteobacteria

^a^ 6776-site data, see text. All alignments available upon request

^b^ Heavily biassed sites removed, all sequences passed chi squared test for amino acid equilibrium with Puzzle; 3100-site data, see text.

^c^ Puzzle was performed with eight rate classes and gamma estimated from the data

^d^ not determined (method did not provide a support value)
