# Supplementary material for: Phylogenomics of the Reproductive Parasite Wolbachia pipientis wMel: A Streamlined Genome Overrun by Mobile Genetic Elements
Source: PLoS Biol. 2004 Mar 16;2(3):e69. doi: 10.1371/journal.pbio.0020069 (PMC368164; doi:10.1371/journal.pbio.0020069)
Supplement: Table S8 — (117 KB DOC). [file pbio.0020069.st008.doc]

Table S8. Individual phylogenetic results for *Reclinomonas* mtDNA-encoded proteins.

| Gene | Closest Taxa to Mitochondria | With BP | BP for branch (ric,wol) | Position of *Magnetococcus* & BP | No. of OTUs | No. of sites | Protein variability[d_T_/OTU] |
| --- | --- | --- | --- | --- | --- | --- | --- |
|  |  |  |  |  |  |  |  |
| *rpl2* | ric | 86 | - | n.a. | 45 | 318 | 0.077 |
| *rps7* | ric | 78 | - | n.a. | 46 | 179 | 0.152 |
| *nad10* | wol | 70 | - | x | 34 | 299 | 0.125 |
| *yejV* | wol | 93 | - | n.a. | 16 | 238 | 0.184 |
| *sdh3* | wol | 81 | - | n.a. | 9 | 157 | 0.417 |
| *rps3* | wol | 74 | - | n.a. | 10 | 322 | 0.112 |
| *rps19* | wol | 87 | - | α        65 | 45 | 144 | 0.154 |
| *rps12* | wol | 84 | - | x | 44 | 159 | 0.086 |
| *rpl20* | wol | 82 | - | n.a. | 43 | 140 | 0.212 |
| *rpl11* | wol | 80 | - | α 89 | 44 | 173 | 0.151 |
| *nad4L* | wol | 68 | - | αβγ    91 | 29 | 141 | 0.262 |
| *nad2* | wol | 55 | - | α 75 | 45 | 650 | 0.112 |
| *nad11* | wol | 55 | - | α 72 | 24 | 1069 | 0.054 |
| *ymf16* | (ric,wol) | 59 | 73 | α 94 | 34 | 372 | 0.151 |
| *yejU* | (ric,wol) | 81 | 95 | α 93 | 21 | 283 | 0.126 |
| *yejR* | (ric,wol) | 100 | 53 | x | 26 | 885 | 0.053 |
| *rpl34* | (ric,wol) | 81 | 75 | x | 34 | 55 | 0.356 |
| *rpl27* | (ric,wol) | 78 | 82 | α 97 | 45 | 114 | 0.218 |
| *nad8* | (ric,wol) | 84 | 91 | αβγ    84 | 26 | 247 | 0.166 |
| *nad5* | (ric,wol) | 80 | 100 | α     100 | 28 | 894 | 0.049 |
| *cox3* | (ric,wol) | 54 | 98 | n.a. | 44 | 356 | 0.156 |
| *cox11* | (ric,wol) | 79 | 88 | n.a. | 12 | 259 | 0.212 |
| *cob* | (ric,wol) | 78 | 100 | α     100 | 33 | 650 | 0.073 |
| *atp6* | (ric,wol) | 98 | 86 | x | 27 | 400 | 0.205 |
| *rps1* | (b,s,m,b) | 63 | - | α       90 | 18 | 640 | 0.058 |
| *nad6* | (b,s,m,b) | 76 | 98 | α       91 | 23 | 247 | 0.246 |
| *nad4* | (b,s,m,b) | 81 | 89 | α       69 | 40 | 696 | 0.076 |
| *nad1* | (b,s,m,b) | 63 | 94 | αβγ 100 | 36 | 841 | 0.052 |
| *cox1* | b,s,m,b) | 63 | - | n.a. | 47 | 750 | 0.048 |
| *atp1* | (b,s,m,b) | 84 | 94 | α       48 | 38 | 600 | 0.038 |
| *nad3* | other α | 88 | - | αβγ   85 | 31 | 199 | 0.234 |
| *nad7* | other α | 100 | 66 | x | 30 | 460 | 0.077 |
| *nad9* | other α | 86 | 75 | αβγ    97 | 26 | 680 | 0.086 |
| *rpl14* | other α | 53 | - | α        53 | 44 | 145 | 0.124 |
| *rpl16* | other α | 67 | - | n.a. | 45 | 150 | 0.162 |
| *sdh4* | other α | * | 66 | n.a. | 7 | 144 | 0.532 |
| *tufA* | other α | 43 | - | α       98 | 54 | 412 | 0.031 |
| *rps14* | other α | 73 | - | n.a. | 38 | 120 | 0.279 |
| *cox2* | other α | 77 | - | n.a. | 22 | 487 | 0.141 |
| *atp9* | other α | 91 | 99 | x | 13 | 103 | 0.481 |
| *atp3* | other α | 44 | 67 | α     100 | 38 | 367 | 0.135 |
| *rps4* | other α | 96 | 54 | n.a. | 44 | 265 | 0.146 |
| *yejW* | not α | 100 | 98 | n.a. | 48 | 350 | 0.296 |
| *secY* | not α | 75 | 75 | n.a. | 19 | 492 | 0.129 |
| *rps8* | not α |  85 | - | x | 35 | 142 | 0.266 |
| *rps2* | not α | 82 | 98 | x | 43 | 387 | 0.097 |
| *rps10* | not α |  78 | - | n.a. | 39 | 131 | 0.187 |
| *rpoA* | not α |  72 | 90 | α       95 | 43 | 436 | 0.098 |
| *rpl5* | not α | 46 | 97 | α       72 | 46 | 209 | 0.130 |
| *rpl32* | not α | 95 | - | n.a. | 7 | 73 | 0.804 |

**Notes and abbreviations for Table S5b.**

- only α-proteobacteria in the alignment
- ric: the group (*Rickettsia conorii,Rickettsia prowazekii*)
- wol: *Wolbachia*
- (ric,wol): (*Rickettsia conorii,Rickettsia prowazekii*, *Wolbachia*) in any order
- (b,s,m,b): (*Bradyrhizobium, Sinorhizobium, Mesorhizobium, Brucella*) in any order
- other α : *Reclinomonas* sister to alpha(s), but not to ric, wol, (ric,wol), or (b,s,m,b)
- not α : *Reclinomonas* not specifically sister to α-proteobacteria
- n.a. Not applicable, *Magnetococcus* not in the alignment (no good homologue found)
- x *Magnetococcus* in the alignment, but does not branch with α-proteobacteria
- α (number) *Magnetococcus* branches with α-proteobacteria at the BP given
- α (number) *Magnetococcus* branches with α−proteobacteria at the BP given
- αβγ (number) *Magnetococcus* branches in a group bearing α-, β-, and γ-proteobacteria at BP given
- BP values indicate RELL bootstrap proportions
- *d*_T_/OTU: Total length of the ProtML tree (expressed as substitutions per site) divided by the number of sequences in the tree.

For the groupings found in the analyses of individual genes, the average BP for the position of mitochondria is summarized as follows:

group avg BP no. values

———— ———— —————

ric or wol 76.1 13

(ric,wol) 79.2 11

b,s,m,b 71.7 5

other α 74.3 12

not α 79.1 8
