# Supplementary material for: Phylogenomics of the Reproductive Parasite Wolbachia pipientis wMel: A Streamlined Genome Overrun by Mobile Genetic Elements
Source: PLoS Biol. 2004 Mar 16;2(3):e69. doi: 10.1371/journal.pbio.0020069 (PMC368164; doi:10.1371/journal.pbio.0020069)
Supplement: Table S9 — (47 KB DOC). [file pbio.0020069.st009.doc]

# Table S9. PCR Primers

| Primer Use | Primer Name | Primer Sequence |
| --- | --- | --- |
|  | | |
| cDNA synthesis | | |
|  | WspBR | 5' GCTTTGCTGGCAAAATGG 3' |
|  | WD0817R | 5' TACCAGCCAGAGTTGCTT 3' |
|  | WD0853R | 5' TCAAGTGATGTGTTATCCCGCA 3' |
|  | WD0852R | 5' CCGTAAATACGTTCCAAG 3' |
|  | | |
| Operon virB3-WD0853 confirmation | | |
|  | (virB3-B4)F: | 5' TTATGATATTGCTTAACGTTGTGTGATG 3' |
|  | (virB3-B4)R: | 5' AATGCCGGAGATGAAATGCTTCTTAG 3' |
|  | (virB6-WD0856)F: | 5' CACTTGGCTCAGGTCAGACACCTG 3' |
|  | (virB6-WD0856)R: | 5' TTATGTTGAACCCGCCTTTGTCATT 3' |
|  | (WD0856-855)F: | 5' TTGGGCCTGGGAGAAA 3' |
|  | (WD0856-855)R: | 5' TAGCTCGGCGTAAATCGAACA 3' |
|  | (WD0854-853)F: | 5' CCGGATAGATTAGCCGGAGG 3' |
|  | (WD0854-853)R: | 5' TCAAGTGATGTGTTATCCCGCA 3' |
|  | | |
| Operon virB8-wspB confirmation | | |
|  | (virB8-B9)F: | 5' AACGATGCTCTTCAGGTGAGATT 3' |
|  | (virB8-B9)R: | 5' TCAACTTCACCATCTTCA 3' |
|  | (virB9-B11)F: | 5' TGGCAATTTAAACGCATC 3' |
|  | (virB9-B11)R: | 5' CCCCCACCGGTTCTTT 3' |
|  | (virB11-D4)F: | 5' CCTGGAAATTGTAAATCAACTGTTAGAA 3' |
|  | (virB11-D4)R: | 5' ATATAACTCTGGATGATGCCAACAATA 3' |
|  | (virD4-wspB)F: | 5' TTTGCAAGTGAAACAGAAGG 3' |
|  | (virD4-wspB)R: | 5' TCCTCATCTTCCTCGTCA 3' |
|  | | |
| Co-expression of virB4, virB6 WD0855, and WD0854 | | |
|  | virB4-B6 F1: | 5’-TTATGATATTGCTTAACGTTGTGTGATG-3’, |
|  | virB4-B6 R1: | 5’-GCAGCAGCACCAGCAA-3’, |
|  | virB4-B6 F2: | 5’-CTATATTTTATCAGAAGGTAAAAAATG-3’, |
|  | virB4-B6 R2: | 5’-ATAGAAGTTACGGGAATAGGAGCTAA-3’; |
|  | WD0855-WD0854 F1: | 5’-ACATGGCTCGCTGTTCG-3’, |
|  | WD0855-WD0854 R1: | 5’-GCCAGTCAAAGGCGATG-3’, |
|  | WD0855-WD0854 F2: | 5’-AGGCTAAATTACCTAACACGCCAAAG-3’, |
|  | WD0855-WD0854 R2: | 5’-TCCAAGATGTCCATGCACCATTAACT-3’ |
